# Disruption of psychostimulant-associated memories by single, low dose ketamine in rats

**DOI:** 10.1016/j.neuropharm.2026.110912

**Published:** 2026-03-11

**Authors:** Angela E. Gonzalez, Jonathan D. Ramos, Zada Anderson, Christyne M. Sewell, Shaghayegh Modaberi, Shane T. Hentges, Travis E. Brown, Barbara A. Sorg

**Affiliations:** a Program in Neuroscience, Washington State University, Vancouver, WA, USA; b R.S. Dow Neurobiology, Legacy Research Institute, Portland, OR, USA; c Integrative Physiology and Neuroscience, Washington State University, Pullman, WA, USA

**Keywords:** Cocaine, Fentanyl, Ketamine, Memory, Methamphetamine, Medial prefrontal cortex, Reconsolidation, Substance use disorders

## Abstract

Cocaine and methamphetamine use disorders pose public health concerns due to the high propensity for relapse driven by persistent drug-associated memories. Output from the medial prefrontal cortex (mPFC) regulates relapse and is controlled by parvalbumin (PV) interneurons, which are surrounded by perineuronal nets (PNNs) that support precise PV neuron firing. We previously showed that removal of mPFC PNNs, which reduces PV firing, disrupted reconsolidation of a cocaine self-administration memory when rats were given a novel, but not familiar, memory retrieval session. Ketamine rapidly suppresses PV neuron output, so we tested whether a single low dose (6 mg/kg) administered before or after a familiar or novel memory retrieval session altered cocaine cue reinstatement. Ketamine given prior to the novel, but not familiar, retrieval session reduced cue reinstatement in male Sprague-Dawley rats. In addition, both ketamine treatments combined with a novel retrieval session reduced PNN intensity around PV neurons after cue reinstatement, suggesting that PNN decreases may be unrelated to reinstatement. However, ketamine reduced the number of c-Fos-activated PV neurons immediately after the novel memory retrieval session, indicating that ketamine may reduce reinstatement through reduced PV neuron function at the time of memory retrieval. Ketamine given just prior to a novel memory retrieval session also reduced cue-induced reinstatement in rats trained for methamphetamine, but not fentanyl or sucrose self-administration. Collectively, these studies show that a single, low dose of ketamine combined with a novel memory retrieval reduces psychostimulant-seeking behavior, and may help delineate the mechanisms for ketamine-reduced relapse in psychostimulant use disorders.

## 1. Introduction

Substance use disorders, especially those associated with repeated psychostimulants such as cocaine and methamphetamine, remain significant public health challenges with no FDA-approved treatment. A crucial aspect of this challenge is the persistence of strong drug-associated memories, which maintain drug-seeking behavior and contribute to relapse (Milton and Everitt, 2010). These memories are reinforced through repeated drug-cue pairings and instrumental associations, such as lever pressing in rodent models of drug self-administration.

Memory is thought to be maintained through reconsolidation, which enables memories to update and stay flexible as conditions change (Tronson and Taylor, 2007). Reconsolidation occurs when an existing memory is retrieved and becomes temporarily open to modifications before restabilizing into long-term memory. Memory destabilization is dependent on prediction error (Pedreira et al., 2004; Sevenster et al., 2014), a violation of an expected outcome that conflicts with the actual outcome of an event. During the state of lability (Nader et al., 2000), the memory can be influenced by pharmacological agents or new information to update, alter, or disrupt the expression of a particular memory (Lee et al., 2017). Targeting reconsolidation has proved to be advantageous for impairing expression of various memories, including fear (Nader et al., 2000; Sevenster et al., 2014), appetitive (Exton-McGuinness and Lee, 2015b; Piva et al., 2020), and weaker drug-associated memories (Brown et al., 2008; Slaker et al., 2015). However, well-formed drug self-administration memories appear to be resistant to reconsolidation disruption by typical pharmacological or memory retrieval conditions, known as “boundary conditions.” To counter this resistance to memory disruption, a novel or unpredictable component can be presented at the time of memory retrieval to reduce subsequent expression of memory (Elsey and Kindt, 2017; Exton-McGuinness et al., 2015; Fernandez et al., 2016), suggesting that a dual approach may be needed to disrupt persistent drug-associated memories. For example, the NMDA receptor antagonist, MK801, effectively disrupted reconsolidation of weaker cocaine conditioned place preference memories (Brown et al., 2008), but failed to do so for cocaine self-administration memories (Brown et al., 2008; Exton-McGuinness and Lee, 2015b) unless a novel or unpredictable component was incorporated during memory retrieval to induce a prediction error (Exton-McGuinness and Lee, 2015b). Similarly, we found that enzymatic disruption of perineuronal nets (PNN) in the mPFC impaired the reconsolidation of a weaker cocaine CPP memory (Slaker et al., 2015), but not the reconsolidation of a stronger cocaine self-administration memory, unless PNN disruption was combined with a novel component during the memory retrieval stage (Wingert et al., 2024).

Ketamine is an NMDA receptor antagonist and dissociative anesthetic with rapid-acting and long-lasting antidepressant properties (Ballard and Zarate, 2020; Hess et al., 2022) that may impair memory reconsolidation (Fattore et al., 2018). Although ketamine treatment has promising impact on reducing relapse in human substance use disorders (SUDs) and animal models of SUDs (Dakwar et al., 2017, 2019b; Das et al., 2019; Maltbie et al., 2019; McKendrick et al., 2020; Sun et al., 2024; Suzuki et al., 2000; Thomas and Chambers, 2025; Zhai et al., 2008), relatively unexplored is how to *optimize* the timing of ketamine treatment while *minimizing* its exposure in the treatment of SUDs. Ketamine’s acute and long-lasting antidepressant effects appear to be primarily linked to its role in re-establishing glutamatergic homeostasis within the medial prefrontal cortex (mPFC) (Moghaddam et al., 1997; Xu et al., 2021), which plays a critical role in cocaine-seeking behavior in humans (Childress et al., 1999; Grant et al., 1996; Maas et al., 1998; Volkow et al., 1991; Zhai et al., 2021) and reinstatement behavior in rodents (Capriles et al., 2003; Fuchs et al., 2007; Ma et al., 2014; McFarland and Kalivas, 2001; Park et al., 2002).

Within the mPFC, GABAergic parvalbumin (PV) interneurons, a majority of which are ensheathed in perineuronal nets (PNNs) are integral for maintaining excitatory:inhibitory balance and modulating excitatory output (Ferguson and Gao, 2018; Wingert and Sorg, 2021). PNNs are specialized extracellular matrix structures, and their expression is critical to experience-dependent plasticity (Slaker et al., 2018a). We have discovered that PNNs in the mPFC are significant contributors to the maintenance of cocaine memories (Slaker et al., 2015; Wingert et al., 2024). Moreover, the expression of PNNs (Matuszko et al., 2017; Venturino et al., 2021b) and PV (Zhou et al., 2015) are both susceptible to the effects of ketamine and may contribute to the maintenance of cocaine memories.

Given our prior findings implicating mPFC circuit function in cocaine memory reconsolidation and the direct effects of ketamine on PV neurons, we investigated whether a single, low-dose ketamine in conjunction with a novel retrieval session would impair cocaine memory reconsolidation and reduce cocaine-seeking behavior in self-administering rats. We further examined the levels of PNNs, PV, and the immediate early genes c-Fos and Neuronal PAS domain Protein 4 (Npas4) in the mPFC after cocaine cue-induced reinstatement. Our findings suggest that ketamine may reduce cocaine-associated memory in part through reduced PV neuron function at the time of memory retrieval, which may alter mPFC network stability to interfere with maintenance of cocaine memory. The same dose of ketamine given prior to a novel memory retrieval session also reduced methamphetamine-, but not fentanyl- or sucrose-associated cue reinstatement. Overall, our findings suggest that optimizing ketamine treatment prior to a novel retrieval condition may reduce relapse in psychostimulant use disorders.

## 2. Materials and methods

### 2.1. Animals

A total of 124 male Sprague-Dawley rats (Envigo, Livermore, CA) weighing 275–300 g was used for analysis in this study. Upon arrival, animals were habituated to housing conditions for one week prior to surgery. Animals were initially pair-housed during habituation, and singly housed following surgery in a temperature and humidity-controlled room. Food and water were available *ad libitum*, except while in the self-administration behavioral apparatus. All animals were housed on a 12 h reverse light-dark cycle (lights off at 0730) and experiments were conducted during the dark cycle. Experiments were approved by the Institutional Animal Care and Use Committees at Legacy Research Institute and Washington State University, in accordance with the National Institute of Health’s Guide for the Care and Use of Laboratory Animals. Every effort was made to reduce the amount of pain and suffering, and the number of animals used in this study.

### 2.2. Surgery

Animals weighing ~300 g were anesthetized with ketamine (VedCo)/dexmetetomidine (Orion) 75 mg/kg in saline; intraperitoneal (i.p.). Either a house-made catheter measuring 12 cm in length contained a silicon bead at 2.9 cm from the distal end, or a pre-made catheter measuring 15 cm in length containing 2 silicon collars 2.9 cm from the distal end (Instech, Plymouth, PA) was implanted into the right jugular vein and was secured around the silicon bead or collars. The proximal end of the catheter was subcutaneously fed around the right side of the neck and protruded between the shoulder blades. The catheter length was then adjusted and secured to a harness comprising a house-made, 3D-printed and straps (SAI, Lake Villa, IL). Anesthesia reversal agent Antisedan (Atipamezole, 4 mL/kg, Zoetis) was administered once surgery was complete. Pain management was provided with once-daily subcutaneous injections of Meloxicam (1 mL/kg, Covetrus) over 3 days. Post-surgery antibiotic Rimadyl/Baytril tablets (5 mg, BioServ) were administered in MediGel Sucralose cups (Animal Specialties) once daily for 3 days. Animals recovered for one week prior to start of experiment. Catheter patency was maintained daily with 0.1–0.2 mL flushing solution containing 1% heparin (Fresenius Kabi), cefazolin (100 mg/mL, Hikma Farmeceutica) in saline or, over longer periods of time, with locking solution containing 50% heparin and 50% glycerol (Sigma-Aldrich). Patency was tested weekly with intravenous 0.1–0.2 mL brevital sodium (Par Pharmaceuticals, 0.1%) in saline.

### 2.3. Drugs

Cocaine-HCl (gift from the National Institutes of Health) was dissolved in sterile saline. Ketamine-HCl (VedCo) was diluted in saline (6, 20, or 50 mg/kg delivered at 1 mL/kg) dose or saline (1 mL/kg) was administered i.p. (+)-Methamphetamine-HCl (Millipore-Sigma) was dissolved in sterile saline. Fentanyl citrate (McKesson, Wilsonville, OR) was diluted in sterile saline.

### 2.4. Self-administration

**Fig. 1.**
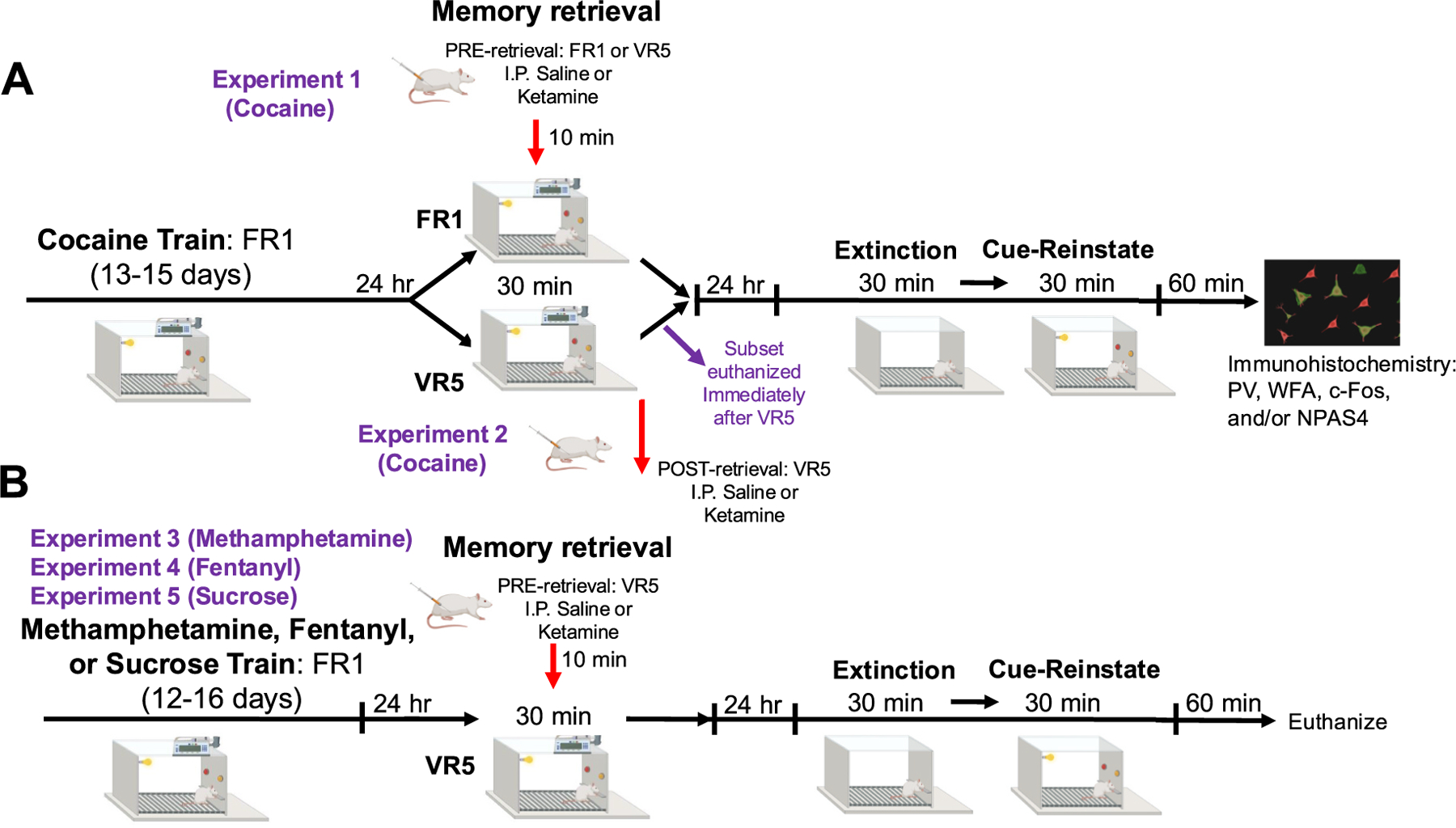
Experimental timelines. Rats were trained as outlined in the Methods in five separate experiments. **(A)** Experiment 1–2 delivering ketamine prior to or after the familiar (FR1) or novel (VR5) retrieval session in cocaine self-administering rats. **(B)** Experiments 3–5 delivered ketamine prior to the novel VR5 retrieval session in methamphetamine, fentanyl, or sucrose self-administering rats.

Self-administration experiments were conducted at the same time of day at the beginning of the animals’ dark cycle between 0800 and 1100 h. Self-administration chambers (Med Associates, Fairfax, VT) contained both active and inactive levers equipped with cue lights above each lever. The chamber also contained a house light. An active lever press resulted in an intravenous reward delivery of 0.5 mg/kg/0.1 mL infusion of cocaine, 0.1 mg/kg/0.1 mL infusion of methamphetamine, or 2 μg/kg/0.1 mL infusion of fentanyl, or sucrose pellets (45 mg, Bio-Serv, Flemington, NJ). Drugs were infused over a 6 s period during which the cue light was activated for the duration of drug delivery, and the cue light was also on for 6 s during pellet delivery. Simultaneously, the house light was activated from the start of the infusion or pellet delivery. The house light indicated a 20 s time-out period during which additional lever presses would not result in additional drug infusions or sucrose pellets. Active lever presses during the 20 s time-out or inactive lever presses did not incur consequences. Active and inactive lever presses were recorded using Med Associates software. Fig. 1 shows the timeline for self-administration Experiments 1–5. Animals were trained to self-administer drug for 2 h/day on a fixed ratio-one (FR1) schedule of reinforcement (one lever press = one cocaine infusion). For cocaine, animals were trained for 13–15 days. They were required to meet criteria of active lever pressing of at least 10 drug rewards on the last 3 days of self-administration. For methamphetamine (Experiment 3), animals were trained for 12 days, and for fentanyl (Experiment 4), animals were trained for 13–16 days. In both the latter two groups, animals needed at least 5 drug rewards and on the last 3 days, and for all drug groups, the number of drug rewards could not decrease sequentially over the last 3 days. For sucrose self-administration (Experiment 5), animals were trained for 13 days, which yielded stable responding defined as ≥ 50 unflavored sucrose pellets for five consecutive days with performance plateauing. Additionally, to help acclimate animals to the upcoming saline or ketamine injection, they were habituated to i.p. saline injections 10 min prior to being placed into the self-administration chambers on the last 3 days of self-administration training.

Twenty-four hours after the last training day, in Experiment 1, animals were given a 30 min cocaine-reinforced retrieval session during which the scheduled reinforcement remained the same (FR1) or changed to a novel schedule of reinforcement (variable ratio-5; VR5) (Exton-Mcguinness and Lee, 2015a). The VR5 schedule of reinforcement is novel and unpredictable because the animal is required to press from 1 to 9 times, with an average of 5 lever presses to receive a single cocaine infusion. In Experiment 1, 10 min prior to retrieval, rats received either saline or a sub-anesthetic dose of ketamine (6 mg/kg), i.p., in their home cage. This dose was determined after pilot testing several subanesthetic doses (12 – 20 mg/kg, ip) prior to retrieval sessions, resulting in little to no lever-pressing that precluded use of these higher doses prior to the retrieval session (data not shown). Lower doses than 6 mg/kg were not tested, given the commonly reported dose of 10 mg/kg for rat experiments (Radford et al., 2018; Saur et al., 2017; Zanos et al., 2016; Zaytseva et al., 2023). For Experiment 1, behavior was counterbalanced across four treatment groups: FR1 + saline (FR1 Sal; 1 mL/kg, i.p., control), FR1 + ketamine (FR1 Ket; 6 mg/kg, i.p.), VR5 + saline (VR5 Sal as above), or VR5 + ketamine (VR5 Ket). In Experiment 2, an additional four groups of animals were administered either saline or ketamine (6, 20, or 50 mg/kg) immediately *after* a VR5 retrieval session. These higher doses could be tested after memory retrieval because they would not interfere with lever pressing during the retrieval session; doses were based on those from previous experiments that generally ranged from 10 to 50 mg/kg or higher (Becker et al., 2003; Hetzler and Berger, 1984; Hunt et al., 2006) and also based on our hypothesis that higher doses may confer increased ability to promote memory reconsolidation. In Experiment 3, two groups of animals were given either saline or 6 mg/kg ketamine prior to a VR5 retrieval session after methamphetamine self-administration training (VR5 Sal or VR5 Ket); in Experiment 4, two groups were given the same treatment (VR5 Sal or VR5 Ket) after fentanyl self-administration training; and in Experiment 5, two groups (VR5 Sal or VR5 Ket) were given access to the same sucrose pellets used during acquisition. For all experiments, active lever responses continued to produce drug or sucrose pellets on a VR5 schedule.

The following day, all groups from Experiments 1–4, with exception of 8 animals that were euthanized immediately after the VR5 session, underwent a 30 min extinction session followed immediately by a 30 min cue reinstatement session. During the 30 min extinction session, animals had access to both active and inactive levers, but no cue light, house light, infusion, or consequence occurred upon lever pressing. Immediately after the extinction session, the cue reinstatement session was begun with an investigator-initiated cue light stimulus from the lever press, resulting in a single cue light presentation. The remainder of the session was uninterrupted, and the cue light was available on an FR1 schedule, during which active lever pressing resulted in both cue light (6 s) and house light (20 s) presentation as above, without drug infusion. Animals were then euthanized 1.5 h after the start of the 30 min reinstatement session.

### 2.5. Immunohistochemistry

Animals were euthanized with Euthasol^®^ (pentobarbital sodium and phenytoin sodium, Virbac) and transcardially perfused with 4% paraformaldehyde in 0.1 M phosphate buffered saline (PBS), pH 7.4. Brains were removed and stored in 4% paraformaldehyde for 24 h and transferred to 20% sucrose in PBS for 48 h. Brains were then flash-frozen with powdered dry ice and stored at −80 °C until sectioning. Brains were sectioned at 40 μm from approximately 4.0 to 3.2 mm from bregma (Paxinos and Watson, 2007) using a Leica Sliding Microtome HM 430 and stored in 25% ethylene glycol (Sigma-Aldrich), 25% glycerol in PBS at −20 °C until immunostaining.

A subset of 7 animals given cocaine self-administration was not included in immunohistochemistry experiments and instead used for additional behavioral assays not included here. Brain sections were stained using a free-floating protocol. Sections were washed 3 times in PBS for 5 min each and transferred to 1% sodium borohydride for a 30-min incubation during which additional sodium borohydride (Sigma-Aldrich) was pipette transferred every 10 min to prevent tissue from drying. Tissue was then washed 4 times in PBS, and between washes, tissue was gently flushed with additional PBS. Tissue was then incubated for 1 h in 5% normal donkey serum (NDS, Sigma-Aldrich) and 0.25% Triton-X (Sigma-Aldrich) in PBS. After incubation, tissue was transferred for overnight incubation at 4 °C in 2% NDS, 0.25% Triton-X, primary antibodies; 1:1000 goat-anti-parvalbumin (Swant Cat PVG 214, RRID: AB_10000345), 1:500 rabbit-anti-c-fos (Thermo Fisher Scientific Cat MA5–15055, RRID:AB_10984728), and 1:200 mouse-anti- Neuronal Per Arnt Sim (PAS) domain protein 4 (Npas4) (Novus Cat NBP2–59332, RRID:AB_3086782) in PBS. The following day, tissue was washed 3 times in PBS for 5 min each and transferred for 2 h incubation at room temperature in 2% NDS, 0.25% Triton-X, secondary antibodies; 1:500 Alexa Fluor donkey-anti rabbit 488 (Thermo Fisher Scientific Cat A32790, RRID:AB_2762833), 1:500 Alexa Fluor donkey-anti mouse 555 (Thermo Fisher Scientific Cat A-31570, RRID:AB_2536180), 1:500 Alexa Fluor donkey-anti goat 405 (Thermo Fisher Scientific Cat A48259, RRID: AB_2890272) in PBS. Tissue was then washed 3 times for 5 min each and transferred for overnight incubation at 4 °C in 2% NDS, 0.25% TX and 1:200 *Wisteria floribunda* agglutinin (WFA) 647 (Bioworld Technology Cat 21511579) in PBS. The following day, tissue was washed 3 times for 5 min each and mounted onto FrostPlus slides using diluted PBS with 0.15% Triton-X. Slides were then cover-slipped using ProlongGold antifade reagent (Vector Laboratories) and sealed with clear nail polish.

### 2.6. Microscopy and image analysis

The prelimbic mPFC was imaged using a Leica DMI 8 confocal microscope. Standardization of laser intensity, gain, offset, and pinhole settings were determined using a section of tissue in the prelimbic PFC from saline control animals. For all experiments except those euthanized immediately after the memory retrieval step (n = 8), 4–6 images were captured per animal at 40X (1.25 numerical aperture) with oil immersion in a z-stack of 10 μm (10/channel). For rats euthanized immediately after memory retrieval, 3–5 images were captured per animal at 20X (0.7 numerical aperture) in a z-stack of 10 μm (10 stacks/channel). The same settings were maintained for all images. Within groups, raw, greyscale and colored images were then exported as.tif files and summed using ImageJ macro, Pipsqueak Pro^™^ (RewireAI^™^, Portland, OR) and analyzed using Polygon AI^™^ (RewireAI^™^).

Image analysis was conducted by an individual blinded to the treatment conditions. Analysis was semi-automated using a pre-trained AI model for PV, WFA, and c-Fos within Polygon AI^™^ to identify positive-labeled cells, while Npas4 was conducted by hand only. For the AI model, initial selection of cells was done, followed by an individual masked to the experimental treatments reviewing all selections manually and making edits as necessary. Once cells were identified, the background was removed, with the rolling ball setting at 50, and images were colocalized using the colocalization feature within Polygon AI^™^. The resulting.csv data files for single, double, triple, and quadruple labeled cells within each image were exported from Polygon AI^™^ for further analysis of cell count, intensity, and colocalization. These data were then processed using an in-house written script to extract cell mean intensity, cell count, colocalized cells, and corresponding intensities and cell counts.

Because we had some negative intensities due to high background, once key data were extrapolated, a signal-to-noise-ratio (SNR) threshold was set to include representative data for each stain type accordingly. The SNR threshold was set at 0.8 for Npas4, 0.8 for PV, 0.85 for WFA, and 1.0 for c-Fos. Mean cell intensity was then adjusted for each cell type where the absolute value of the minimum intensity was added back to all cells of that cell type. Adjusted intensities were then taken as raw data and subsequently normalized to the FR1 Sal condition. The mean of intensities was averaged across slices for each animal, and individual data points shown in the immunohistochemical results represent the average intensities or cell numbers for a single animal.

### 2.7. Statistics

Statistics for behavior and immunohistochemistry were performed using GraphPad Prism software (V10.1.1) or R Statistical Software (V4.2.3 (R Core Team, 2023);). Behavioral experiments assessing multiple independent experimental conditions were analyzed with a two- or three-way (ANOVA), with repeated measures over days or session time where appropriate. If there was a significant interaction, planned comparisons were conducted using a Šídák’s test. In the case that either ANOVA did not reveal an interaction, main effects were interpreted using a Šídák’s test. Immunohistochemistry and behavioral data were analyzed using a two-way ANOVA or Welch’s *t*-test (in the case of two groups). To simplify visual interpretation of significant differences on the graphs, when only main effects of treatment or memory retrieval were found, the p-values are indicated on the graphs (all statistical results are indicated in the Supplemental Tables). Data were considered significant when p < 0.05.

## 3. Results

### 3.1. Ketamine treatment prior to novel memory retrieval session reduces cue reinstatement in cocaine-trained animals

**Fig. 2.**
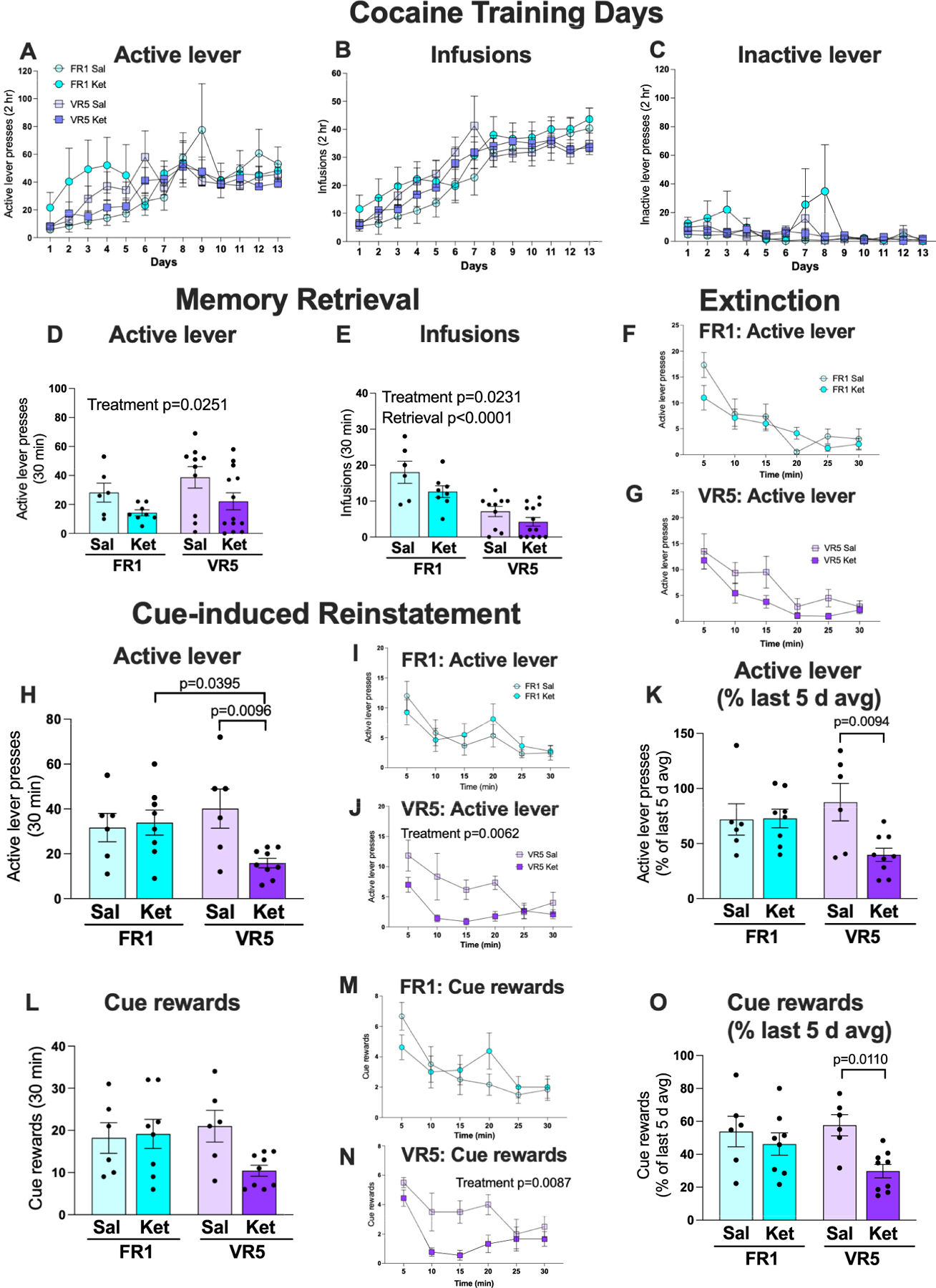
Ketamine given prior to a novel memory retrieval session impairs reinstatement of cocaine-seeking behavior. **(A)** Active lever presses during acquisition of cocaine self-administration. One animal in the VR5 Sal group pressed 1137 times on Day 7 and was omitted in the graph for clarity, but is included in the statistical analysis. There were no significant changes in active lever pressing over time due to variability between rats. (**B**) Number of cocaine infusions during acquisition. (**C**) Number of inactive lever presses during acquisition. (**D, E**) Memory retrieval session for (**D**) active lever presses and (**E**) rewards (cocaine infusions). (**F, G**) Extinction time course for total active lever presses in the (**F**) familiar FR1 group and (**G**) novel VR5 group. (**H**) Cue-induced reinstatement for active lever presses. (**I, J**) Cue-reinstatement timecourse for active lever presses in the (**I**) familiar FR1 group and (**J**) novel VR5 group. (**K**) Within-subjects comparison of active lever presses, expressed as percent of the last 5 day average number of lever presses during training days. (**L**) Cue-induced reinstatement for rewards (cue-light presentations). (**M, N**) Cue-reinstatement timecourse for rewards in the (**M**) familiar FR1 group and (**N**) novel VR5 group. (**O**) Within-subjects comparison of rewards, expressed as percent of the last 5 day average number of lever presses during training days. Data are mean ± SEM.

Previously, our lab (Wingert et al., 2024) and others (Exton-Mcguinness and Lee, 2015a) showed that impairing reconsolidation of cocaine memories in self-administering rats requires a memory retrieval session that introduces a novel cocaine reward contingency. Here we examined whether pretreatment with a single administration of a low-dose ketamine treatment prior to memory retrieval was sufficient to impair subsequent cue-induced cocaine-seeking behavior in cocaine self-administering rats. Fig. 1A (Experiment 1) shows the experimental timeline. The cumulative number of active or inactive lever presses, cocaine infusions or inactive lever presses were not different among the four groups (Supplemental Table 1). Fig. 2A–C shows that all animals trained to the same extent across days. While there were no significant effects across time for active lever presses due to high variability among animals (Fig. 2A), for cocaine infusions, there was a significant day effect (F4.23, 139.54 = 30.49; p < 0.0001), with increasing number of infusions across training. There were no differences in inactive lever presses across days (Fig. 2C).

During the memory retrieval session, ketamine (6 mg/kg, ip) reduced the number of active lever presses, with a main effect of treatment (F1, 33 = 5.510; p = 0.0251) (Fig. 2D). Ketamine also reduced the number of cocaine infusions, with a main effect of treatment (F1, 33 = 5.677; p = 0.0231) and retrieval (F1, 33 = 31.10; p < 0.0001) (Fig. 2E), with decreases in both FR1 (p = 0.0004) and VR5 (p = 0.0016) groups. There were no differences among groups for inactive lever presses (not shown).

The following day, all groups demonstrated extinction during the 30 min session in which no cocaine rewards or cues were obtained (FR1 group: time F3.687, 44.24 = 17.34; p < 0.0001; Fig. 2F) (VR5 group: time F3.532, 45.91 = 14.86; p < 0.0001; Fig. 2G), and no differences in inactive lever presses among groups for FR1 or VR5 retrieval (not shown). However, when the cocaine cue once again became available upon lever press, ketamine reduced cue-induced reinstatement, but only in rats given the novel VR5 retrieval session the day before (treatment × retrieval interaction F1, 25 = 5.5557; p = 0.0265). In the VR5 retrieval group, post-hoc analysis revealed that ketamine decreased lever pressing compared with saline treated rats (p = 0.0096), and also compared with animals given FR1 retrieval and ketamine (p = 0.0395) (Fig. 2H). The time course of cue reinstatement indicates that all groups reduced active lever pressing across time (FR1: p = 0.0025; VR5: p = 0.0011) (Fig. 2I–J). However, VR5 retrieval combined with ketamine rapidly diminished lever pressing behavior during cue reinstatement (treatment F1, 13 = 10.64; p = 0.0062) (Fig. 2J). There were no differences among groups for inactive lever presses (not shown; Supplemental Table 2).

We also assessed lever presses during cue reinstatement as a percent of their maintenance self-administration levels, which were defined as the last 5 days of training. While a three-way ANOVA on training days revealed a retrieval × day interaction (F2.67, 88.11 = 3.118; p = 0.035), post-hoc comparison showed no significant impact of retrieval condition on any of the 5 days (p > 0.05). When normalizing cue responding within subjects, ketamine reduced active lever responding during cue reinstatement (treatment F1, 25 = 4.505; p = 0.0449; treatment x retrieval F1, 25 = 4.878; p = 0.0366), and post-hoc analysis reflected what we observed above showing reduced cocaine cue seeking when ketamine was combined with VR5 retrieval (p = 0.0094) (Fig. 2K).

The total number of cue rewards obtained was not different after ketamine (treatment × retrieval interaction p = 0.0692) (Fig. 2L). While the time course revealed a significant reduction in the number of cue rewards over time in saline and ketamine treated rats in both retrieval groups (FR1: time F3.166, 37.99 = 6.515; p = 0.0010 (Fig. 2M); (VR5: time F2.976, 38.69 = 7.891; p = 0.0003) (Fig. 2N), only when ketamine was combined with VR5 retrieval was lever pressing reduced more rapidly (treatment F1, 13 = 9.512; p = 0.0087) (Fig. 2N). There was no difference in cue rewards between groups over the last 5 days of maintenance responding (p > 0.05), and when normalizing cue responding within subjects, ketamine reduced the number of cue rewards obtained during cue reinstatement (treatment F1, 25 = 7.280; p = 0.0123), and post-hoc analysis for VR5 retrieval showed a reduced number of rewards in the ketamine group (p = 0.0110) (Fig. 2O). These findings suggest that ketamine suppressed the reconsolidation of a cocaine-associated cue memory, but only when it was combined with a *novel contingency* during memory retrieval. All statistical results for Fig. 2 are shown in Supplemental Table 2.

### 3.2. Ketamine given immediately after a novel retrieval session does not impair cue reinstatement in cocaine-trained animals

**Fig. 3.**
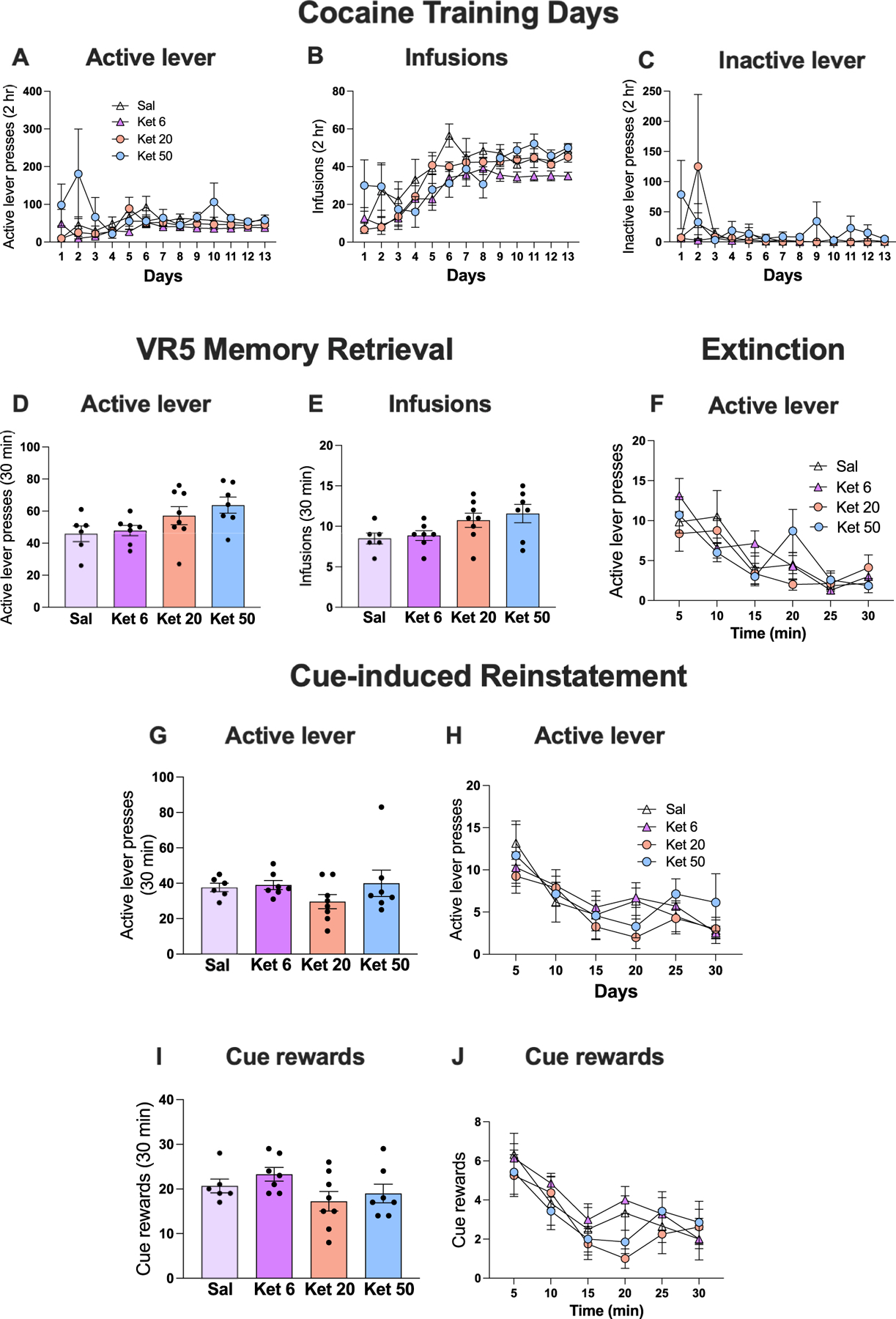
Ketamine given after a novel memory retrieval session does not alter reinstatement of cocaine-seeking behavior. **(A)** Active lever presses during acquisition of cocaine self-administration. (**B**) Number of cocaine infusions during acquisition. (**C**) Number of inactive lever presses during acquisition. (**D, E**) Memory retrieval session for (**D**) active lever presses and (**E**) rewards (cocaine infusions). (**F**) Extinction time course for total active lever presses in all groups. (**G**) Cue-induced reinstatement for active lever presses. (**H**) Cue-reinstatement timecourse for active lever presses in all groups. (**I**) Cue-induced reinstatement for rewards (cue-light presentations). (**J**) Cue-reinstatement timecourse for rewards in all groups. Data are mean ± SEM.

We previously found that enzymatic removal of PNNs prior to, but not after, a VR5 novel retrieval session reduced cocaine cue reinstatement (Wingert et al., 2024). Therefore, we tested whether ketamine given immediately *after* retrieval would reduce subsequent cue reinstatement. Fig. 1A (*Experiment 2*) shows the experimental timeline. We focused our studies on delivery of the novel VR5 memory retrieval session, given the absence of ketamine effects when combined with the familiar FR1 retrieval session. In separate cohorts, we tested the impact of the same dose of ketamine (6 mg/kg) in addition to two higher doses (20 or 50 mg/kg) on subsequent cue-induced reinstatement. The cumulative number of active lever presses, cocaine infusions, or inactive lever presses were not different among groups (Supplemental Table 1). Fig. 3A shows active lever presses across training days, and while there was a treatment × day interaction (F36, 288 = 1.492; p = 0.0404), there were no differences from the saline control group. The number of cocaine infusions increased over days (day F4.416, 106.0 = 21.24; p < 0.0001) and there was a treatment × day interaction (F36, 288 = 1.702; p = 0.0097), with a decrease in the 6 mg/kg ketamine group compared with the saline group on days 6 (p = 0.0460) and 13 (p = 0.0086) (Fig. 3B). No differences among groups were found for inactive lever presses across training days (not shown; Supplemental Table 3).

Despite the differences in cue rewards during acquisition of cocaine self-administration training, there were no differences among groups in the number of active levers or cocaine infusions on the memory retrieval session (Fig. 3D and E). For inactive lever presses, there was a treatment effect (p = 0.0144), however, no groups to be given ketamine after retrieval were different from saline controls (not shown; Supplemental Table 3).

The following day, all groups demonstrated extinction during the 30 min session in which no cocaine rewards or cues were obtained, with no differences in active lever presses between saline or ketamine groups (time F3.792, 91.01 = 17.18; p < 0.0001; Fig. 3F). In contrast to when ketamine was given *prior to* VR5 memory retrieval, none of the doses of ketamine given just *after* VR5 retrieval altered cue reinstatement (Fig. 3G–J), with only a time effect for both active and cue rewards (p < 0.0001) and no differences in inactive lever presses (not shown; Supplemental Table 3). Here we did not assess normalized behavior on the cue day (expressed as a percentage of their maintenance responding) because, while counterbalanced for cumulative lever presses and rewards (Supplementary Table 1), we found a main treatment effect for active lever presses on the last 5 days of training (treatment F3, 24 = 4.835; p = 0.009) and cue rewards (F3, 24 = 9.728; p = 0.0002). Overall, ketamine given immediately post-VR5 retrieval did not impair cocaine cue reinstatement as was observed for pre-VR5 retrieval ketamine treatment, suggesting that ketamine-induced changes in the circuit *at the time of memory retrieval* shapes future cue-induced drug-seeking behavior. All statistical results for Fig. 3 are shown in Supplemental Table 3.

### 3.3. Ketamine treatment given either prior to or after novel memory retrieval session reduces PNN intensity following cue reinstatement in cocaine-trained animals

**Fig. 4.**
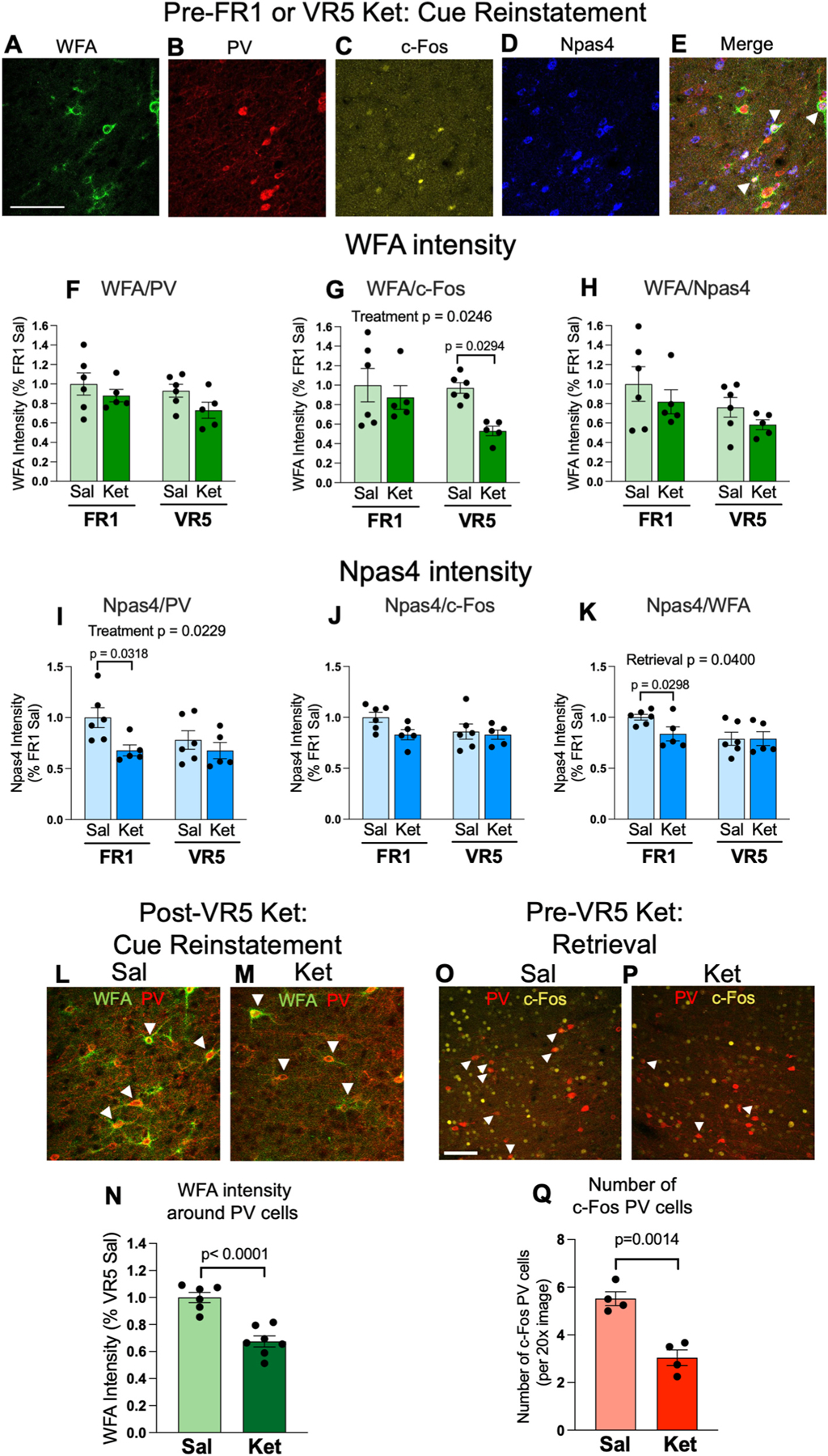
mPFC PNNs and c-Fos activated PV cells are reduced by ketamine combined with novel memory retrieval session. (**A-D**) Representative images of mPFC (**A**) WFA, (**B**) PV, (**C**) c-Fos, (**D**) Npas4, and (**E**) Merged (quadruple-labeled cells); white arrows in merged image indicate quadruple-labeled cells. Scale bar = 100 μm. **(F-H**) WFA staining intensity expressed as a percent of FR1 Sal controls for WFA-surrounded (**F**) PV cells, (**G**) c-Fos cells, and (**H**) Npas4 cells. (**I-K**) Npas4 intensity in cells co-labeled with (**I**) PV, (**J**) c-Fos, and (**K**) WFA. (**L, M**) Representative mPFC confocal images of WFA/PV cells after cue reinstatement in animals given post-VR5 ketamine in (**L**) Sal and (**M**) Ket treated rats; white arrows indicate WFA/PV double-labeled cells. (**N**) WFA intensity around PV cells after cue reinstatement in the same groups. (**O, P**) Representative mPFC confocal images of c-Fos-containing PV cells after the retrieval session in (**O**) Sal and (**P**) Ket treated rats; white arrows indicate c-Fos/PV double-labeled cells. Scale bar = 100 μm. (**Q**) Number of c-Fos-containing PV cells after the retrieval session in the same groups. Data are mean ± SEM.

PNNs surround a majority of PV interneurons in the mPFC (Slaker et al., 2015) and low-dose ketamine treatment has been shown to selectively target PV cells (Ng et al., 2018). Most mPFC PV neurons contain NMDA receptors (Lewis et al., 2022), and acute ketamine rapidly reduces PV cell activity via NMDA receptor blockade (Picard et al., 2019) and reduces PV levels (Zhou et al., 2015). We therefore examined the mPFC for expression of PNNs, PV, and also assessed c-Fos and Npas4 as markers for activation of cells 1.5 h post-cue-reinstatement. We chose to examine c-Fos because of its widespread activation in the brain after environmental stimuli (Bijoch et al., 2023), and Npas4, because relatively few studies have examined this transcription factor in the mPFC and the role it may play in reconsolidation. Like c-Fos, its expression is also activity dependent, but it is activated exclusively in neurons by calcium influx in cells that do not always overlap c-Fos-activated neurons (Sun et al., 2020). Npas4 also regulates inhibitory synapses on excitatory neurons (Lin et al., 2008) and contributes to memory reactivation (Ploski et al., 2011). Fig. 4A–E shows images of these markers in the prelimbic mPFC. We analyzed both the intensity (Table 4, Supplemental Table 4) and the number of cells containing the markers (Supplemental Fig. 1, Supplemental Table 5). While neither ketamine nor retrieval condition altered the intensity of the PNN marker WFA around PV cells (Fig. 4F), PNN intensity around c-Fos-containing cells was reduced by the combination of ketamine and the novel VR5 retrieval (treatment F1, 18 = 6.018; p = 0.0246); this reduction was driven by the decrease after VR5 retrieval (p = 0.0294) (Fig. 4G). The intensity of WFA surrounding Npas4 cells was not altered (Fig. 4H). When ketamine was given post-retrieval, PNN intensity around PV cells was reduced (Fig. 4L–N), showing that the impact of ketamine on PNN intensity levels after cocaine cue reinstatement is likely independent of when it is given relative to the novel VR5 retrieval session.

Ketamine generally reduced Npas4 intensity in PV cells (treatment F1, 18 = 6.185; p = 0.0229); this reduction was driven by a decrease in intensity after FR1 retrieval (p = 0.0318) (Fig. 4I). In addition, the novel VR5 session reduced Npas4 intensity in PNN-surrounded cells (retrieval F1, 18 = 4.901; p = 0.0400), and ketamine decreased Npas4 intensity in the FR1 (p = 0.0298) but not in the VR5 group (Fig. 4K). We also found no changes in Npas4 intensity in c-Fos-containing cells (Fig. 4J). We found no changes in the intensity of PV cells co-localized with PNNs, c-Fos, or Npas4 (Supplemental Fig. 1A–C) or in the intensity of c-Fos cells co-localized with PNNs, PV or Npas4 (Supplemental Fig. 1D–F). No changes were found in the *number* of cells co-stained for these markers (Not shown; Supplemental Table 5). These findings collectively show that ketamine combined with the novel VR5 retrieval session decreased PNN intensity independent of whether ketamine was administered prior to or immediately after the VR5 memory retrieval session. The decrease in Npas4 intensity by ketamine and/or VR5 retrieval suggests that this protein is susceptible to either manipulation. All statistical results for Fig. 4 are shown in Supplemental Tables 4 and 5

### 3.4. Ketamine treatment prior to novel memory retrieval session reduces number of c-fos-activated PV cells immediately following retrieval in cocaine-trained animals

Since we found decreased PNN intensity after the cue reinstatement session, we assessed whether PNNs or PV cells were impacted differently by ketamine when examined *immediately after* the novel VR5 retrieval session. There were no changes in PNN intensity (Supplemental Table 4). However, we found that ketamine decreased the number of c-Fos-containing PV cells by approximately 45% in the mPFC (p = 0.0014, Fig. 4O–Q). This decrease in c-Fos/PV cells was not reflected in the total number of c-Fos cells (Sal = 138.1 ± 21.0; Ket = 163.3 ± 31.8 cells; p = 0.5321) or in the total number of PV cells (Sal = 26.0 ± 1.1; Ket = 21.2 ± 3.2, p = 0.1998), indicating a rapid effect of ketamine on reducing PV cell activation, which may in turn reduce PNN intensity when assessed 24 h later, given that PV and PNNs may co-regulate each other’s expression (see Discussion). These findings suggest that reduced PV cell function at the time of retrieval may interfere with memory precision and/or the motivation to obtain the cue reward.

### 3.5. Ketamine treatment prior to novel memory retrieval session reduces cue reinstatement in methamphetamine-trained animals

**Fig. 5.**
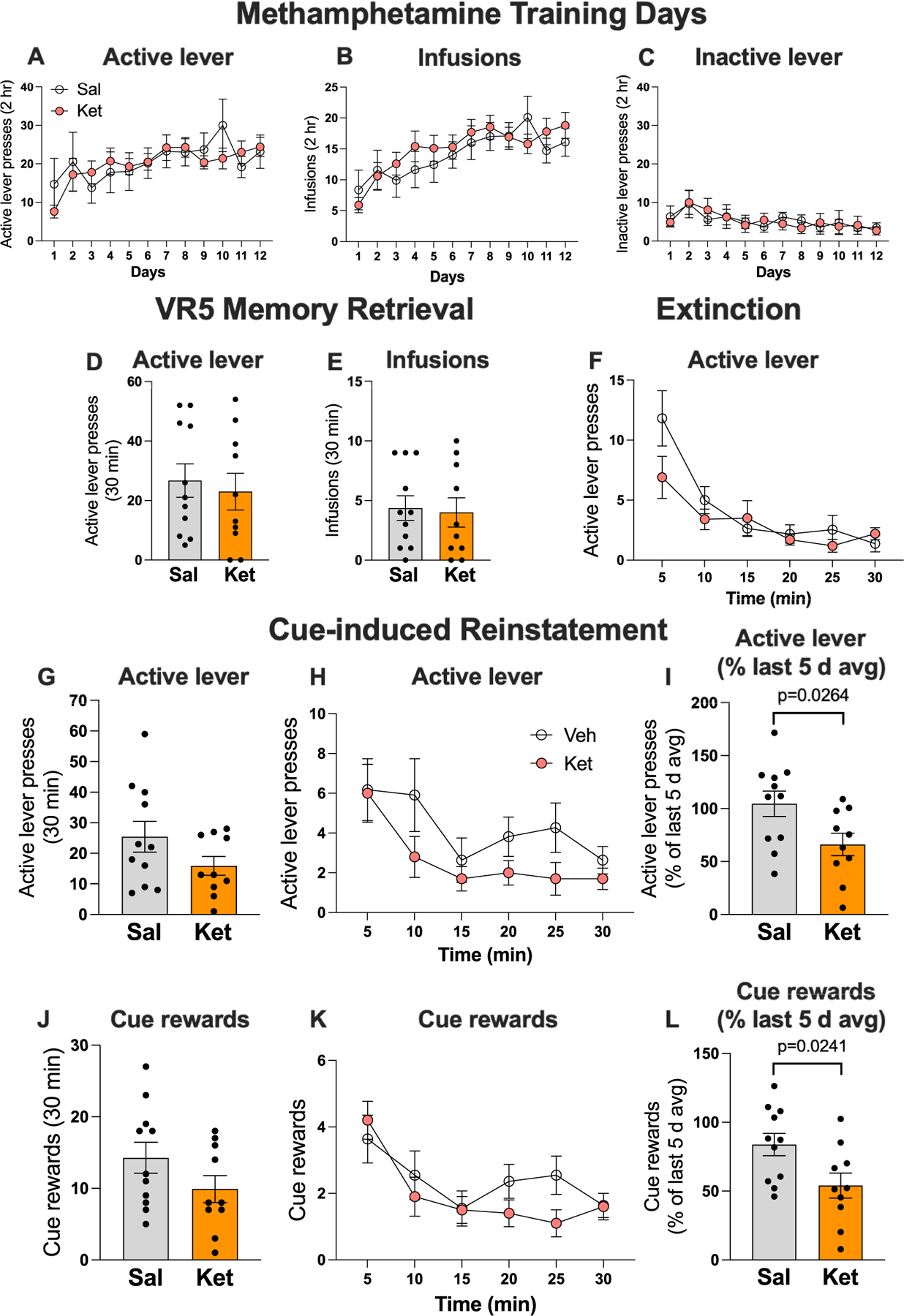
Ketamine prior to novel memory retrieval session impairs reinstatement of methamphetamine-seeking behavior. **(A)** Active lever presses during acquisition of methamphetamine self-administration. (**B**) Number of methamphetamine infusions during acquisition. (**C**) Number of inactive lever presses during acquisition. (**D, E**) Memory retrieval session for (**D**) active lever presses and (**E**) rewards (methamphetamine infusions). (**F**) Extinction time course for total active lever presses in both groups. (**G**) Cue-induced reinstatement for active lever presses. (**H**) Cue-reinstatement timecourse for active lever presses in both groups. (**I**) Within-subjects comparison of active lever presses, expressed as percent of the last 5 day average number of lever presses during training days. (**J**) Cue-induced reinstatement for rewards (cue-light presentations). (**K**) Cue-reinstatement timecourse for rewards in both groups. (**L**) Within-subjects comparison of rewards, expressed as percent of the last 5 day average number of lever presses during training days. Data are mean ± SEM.

Given the reduced cocaine cue reinstatement in animals given ketamine combined with the novel retrieval session, we then assessed whether the same dose of ketamine given prior to the novel VR5 retrieval session would reduce cue reinstatement in rats trained to self-administer another psychostimulant, methamphetamine. Fig. 1B shows the timeline of Experiment 3. The cumulative number of active or inactive lever presses, cocaine infusions or inactive lever presses were not different among the two groups (Supplemental Table 1). Fig. 5A and B shows that both groups of animals trained to the same extent across days (Active lever presses: day F3.701, 70.32 = 4.465; p = 0.0036; Infusions: day F4.283, 81.37 = 11.43; p < 0.0001). There were no differences across treatment or day in inactive lever presses (Fig. 5C). During VR5 memory retrieval, there were no differences between saline and ketamine groups for active lever presses (Fig. 5D) or methamphetamine infusions (Fig. 5E), but there was a decrease in the number of inactive lever presses in the ketamine group (not shown; p = 0.0471). The following day, both groups demonstrated extinction during the 30 min session in which no methamphetamine rewards or cues were obtained, with no differences in active lever presses between saline or ketamine groups (time F2.856, 54.26 = 16.22; p < 0.0001) (Fig. 5F) or for inactive lever presses (time F2.203, 41.85 = 4.552; p = 0.0138) (not shown). Cue reinstatement was not different between groups for the number of active lever presses (Fig. 5G), with a decrease across time in both groups (time F3.141, 59.67 = 7.527; p = 0.0002) (Fig. 5H). There was no difference in active lever presses or cue rewards between groups over the last 5 days of self-administration training (p > 0.05), and thus we again normalized within subjects to examine active lever presses during reinstatement as a percent of their last 5 days of self-administration training. For active levers, ketamine reduced cue reinstatement (p = 0.0264) (Fig. 5I). For cue rewards, while there were no differences in cue reinstatement between groups (Fig. 5J and K), ketamine decreased reinstatement when normalized within animals to their last 5 days of self-administration training (p = 0.0241) (Fig. 5L). There were no differences in inactive lever pressing during cue reinstatement (not shown). These findings demonstrate that ketamine combined with a novel retrieval session reduced cue reinstatement similar to what was found after cocaine self-administration training, although with a reduced magnitude. All statistical results for Fig. 5 are shown in Supplemental Table 6.

### 3.6. Ketamine treatment prior to novel memory retrieval session does not alter cue reinstatement in fentanyl-trained animals

**Fig. 6.**
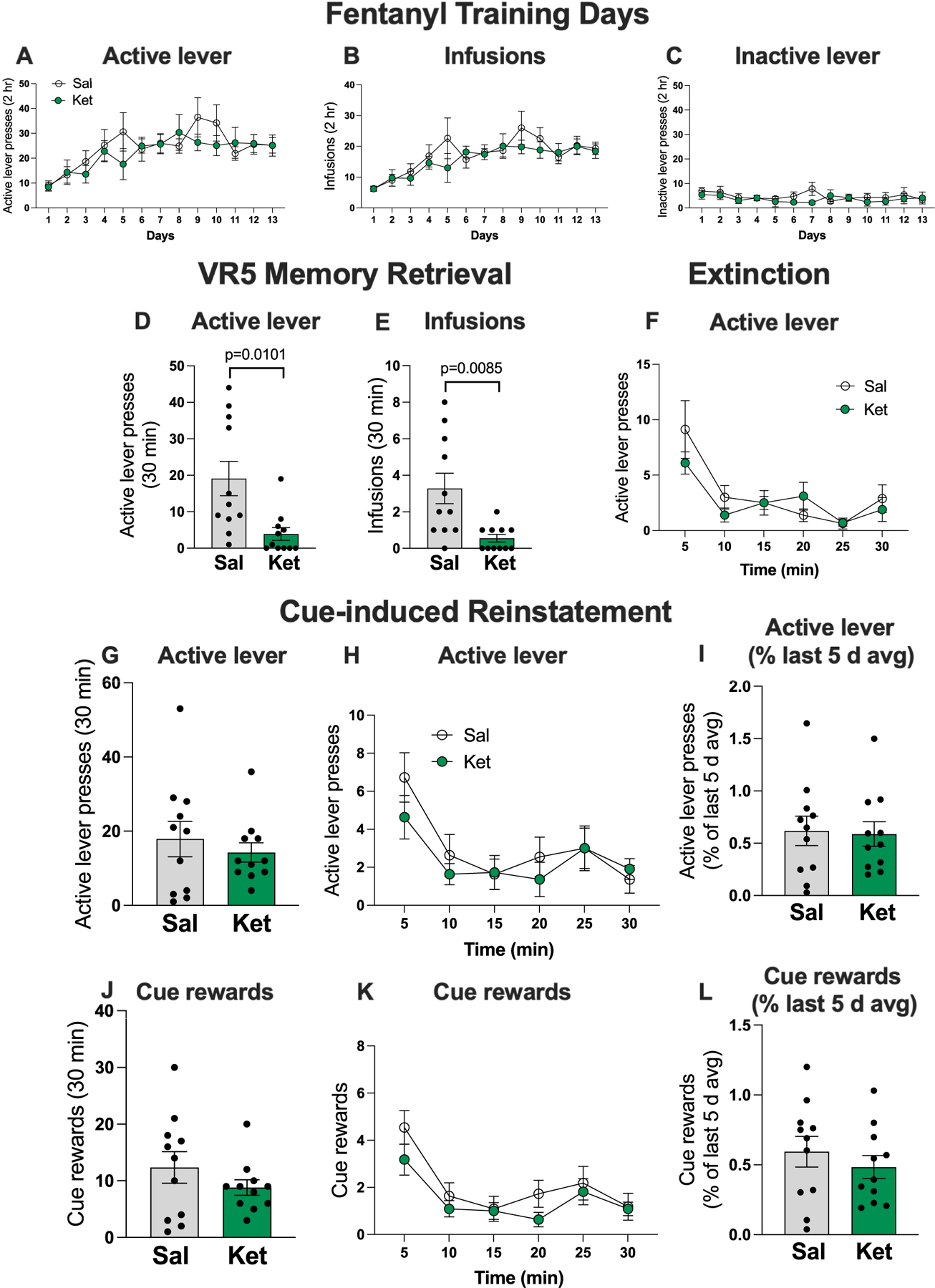
Ketamine prior to novel memory retrieval session does not alter reinstatement of fentanyl-seeking behavior. **(A)** Active lever presses during acquisition of fentanyl self-administration. (**B**) Number of fentanyl infusions during acquisition. (**C**) Number of inactive lever presses during acquisition. During training, days 4 and 5 from one saline animal are missing. In addition, on day 5 of training, data from 5 animals in the saline group and 6 animals in the ketamine group are missing due to malfunction of equipment. (**D, E**) Memory retrieval session for (**D**) active lever presses and (**E**) rewards (fentanyl infusions). (**F**) Extinction time course for total active lever presses in both groups. For the extinction time course data, three animals are missing from the saline group and two animals from the ketamine group due to malfunction of equipment when obtaining the timecourse results. (**G**) Cue-induced reinstatement for active lever presses. (**H**) Cue-reinstatement timecourse for active lever presses in both groups. (**I**) Within-subjects comparison of active lever presses, expressed as percent of the last 5 day average number of lever presses during training days. (**J**) Cue-induced reinstatement for rewards (cue-light presentations). (**K**) Cue-reinstatement timecourse for rewards in both groups. (**L**) Within-subjects comparison of rewards, expressed as percent of the last 5 day average number of lever presses during training days. Data are mean ± SEM.

We next determined whether ketamine given prior to the novel VR5 retrieval session would reduce cue reinstatement in rats trained to self-administer the opioid fentanyl, a different class of drug than cocaine or methamphetamine. Fig. 1B shows the timeline of Experiment 4. The cumulative number of active or inactive lever presses, cocaine infusions or inactive lever presses were not different among the two groups (Supplemental Table 1). Fig. 6A–B shows that both groups of animals trained to the same extent across days (active lever presses: day F5.275, 99.79 = 5.756; p < 0.0001; infusions: day F4.589, 86.82 = 9.210; p < 0.0001). There were no differences in inactive lever presses (Fig. 6C). During VR5 memory retrieval, ketamine reduced both active lever presses (p = 0.0101) (Fig. 6D) and fentanyl infusions (p = 0.0085) (Fig. 6E), with no differences in the number of inactive lever presses (not shown; Supplemental Table 7). During extinction the next day, both groups demonstrated reduced active lever pressing over the session (time F3.359, 57.10 = 13.29, p 0.0001) (Fig. 6F), with no changes in inactive lever presses (not shown; Supplemental Table 7). Despite the decreased responding during the retrieval session the day prior, ketamine did not alter cue reinstatement: there were no differences in active lever presses (Fig. 6G), with decreases in lever presses over time in both groups (time F 3.571, 74.99 = 7.978; p < 0.0001) (Fig. 6H). There were also no differences when reinstatement was normalized to their last 5 days of self-administration training, which was not different between the treatment groups (p > 0.05) (Fig. 6I). Similarly, ketamine did not alter the number of cue rewards during cue reinstatement (Fig. 6J), with decreases in cue rewards obtained over time in both groups (time F 3.726, 78.24 = 12.38; p 0.0001) (Fig. 6K) and no differences when normalized to their last 5 days of self-administration training (Fig. 6L). For inactive lever presses, there were no differences between saline and ketamine groups for inactive lever presses during cue reinstatement (not shown; Supplemental Table 7). These findings show that ketamine combined with the novel VR5 retrieval session was ineffective in reducing cue reinstatement in fentanyl-trained animals. All statistical results for Fig. 6 are shown in Supplemental Table 7.

### 3.7. Ketamine treatment prior to novel memory retrieval session does not alter cue reinstatement in sucrose-trained animals

**Fig. 7.**
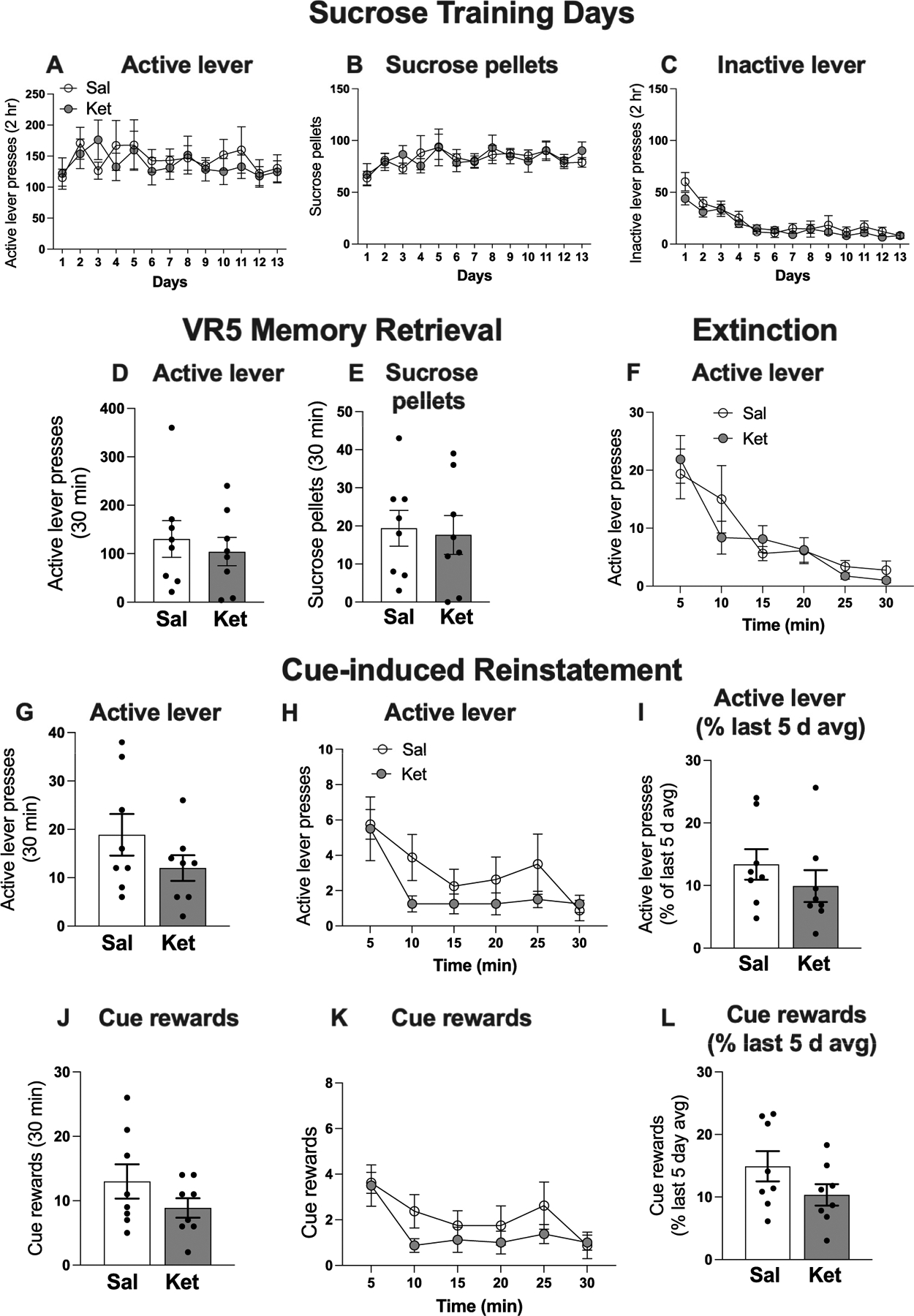
Ketamine prior to novel memory retrieval session does not alter reinstatement of sucrose-seeking behavior. **(A)** Active lever presses during acquisition of sucrose self-administration. (**B**) Number of sucrose pellets during acquisition. (**C**) Number of inactive lever presses during acquisition. (**D, E**) Memory retrieval session for (**D**) active lever presses and (**E**) rewards (sucrose pellets). (**F**) Extinction time course for total active lever presses in both groups. (**G**) Cue-induced reinstatement for active lever presses. (**H**) Cue-reinstatement timecourse for active lever presses in both groups. (**I**) Within-subjects comparison of active lever presses, expressed as percent of the last 5 day average number of lever presses during training days. (**J**) Cue-induced reinstatement for rewards (cue-light presentations). (**K**) Cue-reinstatement timecourse for rewards in both groups. (**L**) Within-subjects comparison of rewards, expressed as percent of the last 5 day average number of lever presses during training days. Data are mean ± SEM.

Given that ketamine reduced cue-induced reinstatement in rats trained for psychostimulant self-administration but not for fentanyl, we also tested whether ketamine would alter cue-induced reinstatement in rats trained for sucrose, a non-drug reward. The cumulative number of active or inactive lever presses, sucrose pellets obtained, or inactive lever presses were not different among the saline and ketamine groups (Supplemental Table 1). Fig. 1B shows the timeline of Experiment 5. Fig. 7A and B shows that both groups of animals rapidly acquired self-administration of sucrose pellets and trained to the same extent across days, with no effect of training day except for inactive lever presses (inactive lever presses: day F4.566, 63.92 = 26.10; p < 0.0001). During the novel VR5 memory retrieval session, there were no differences between ketamine and saline-treated animals for active lever presses or sucrose pellet rewards (Fig. 7D–E, respectively), and no differences in inactive lever presses (data not shown, Supplemental Table 8). During extinction the next day, both saline and ketamine groups demonstrated reduced active lever pressing over the session (time F1.945, 27.22 = 18.70, p < 0.0001) (Fig. 7F), and for inactive lever presses (time F2.464, 34.49 = 18.90; p < 0.0001; not shown). Cue reinstatement was not different between groups for the number of active lever presses (Fig. 7G), with a decrease across time in both groups (time F3.092, 43.29 = 5.938; p = 0.0016) (Fig. 7H). There was no difference in active lever presses or cue rewards between groups over the last 5 days of self-administration training (p > 0.05), and thus we again normalized within subjects to examine active lever presses during reinstatement as a percent of their last 5 days of training. Within-subjects comparison showed that there were no differences in active lever responding for the sucrose cue (Fig. 7I). Similarly, for cue rewards, there were no differences in cue reinstatement between groups (Fig. 7J), with a decrease across time in both groups (time F3.399, 47.59 = 4.835; p = 0.0037 (Fig. 7K) and no within-subject differences in rewards for the sucrose cue (Fig. 7L). There were no differences in inactive lever pressing during cue reinstatement (not shown). These findings demonstrate that ketamine combined with a novel retrieval session was ineffective at altering sucrose-associated cue reinstatement. All statistical results for Fig. 7 are shown in Supplemental Table 8.

## 4. Discussion

These studies demonstrate the ability of a single, low dose ketamine administration to reduce cocaine- and methamphetamine- but not fentanyl- or sucrose-associated cue reinstatement in self-administering rats. From the cocaine experiments, we found that ketamine given *prior to* a memory retrieval session reduced subsequent cue reinstatement, but only when the memory retrieval session included a novel component; in this case, a new contingency to obtain cocaine reward (VR5 session). In contrast, ketamine given immediately *after* the novel memory retrieval session failed to reduce cue reinstatement. Ketamine also reduced the intensity of PNNs in the prelimbic mPFC when examined after cocaine cue reinstatement when ketamine was given either prior to or after the novel retrieval memory session, suggesting that decreased PNN intensity after cue reinstatement was not directly related to reinstatement behavior tested one day later. When we tested the impact of the novel cocaine retrieval session on PNN intensity immediately after *retrieval*, there was no effect. However, the number of c-Fos-containing PV cells was substantially reduced, suggesting that reduced PV neuron activation may be required *at the time of cocaine memory retrieval* to suppress cue reinstatement. Ketamine given prior to a novel memory retrieval session similarly reduced cue reinstatement in animals trained to self-administer methamphetamine, albeit with a reduced magnitude compared with cocaine. In contrast, ketamine given before a novel memory retrieval session did not alter cue reinstatement in animals trained to self-administer fentanyl or sucrose, suggesting a specificity in ketamine’s ability to reduce cue-induced relapse to psychostimulant-associated cues.

### 4.1. Single-use ketamine reduces cocaine cue-induced reinstatement

The reduction in responding for the cocaine cue may be related to the blunting of memory reconsolidation, given that ketamine prior to retrieval of a familiar memory (FR1 retrieval session) did not alter cue reinstatement. Indeed, several observations that well-entrenched memories require a novel component temporally close to the retrieval session or incorporation into the retrieval session itself (Exton-Mcguinness and Lee, 2015a; Fernandez et al., 2016; Wingert et al., 2024) is consistent with our interpretation of reduced reconsolidation of cocaine-associated memory. While we cannot be certain that ketamine did not impair retrieval of the memory associated with cocaine due to reduction in active lever pressing in some animals during retrieval (see below), similar impairment was found in the familiar FR1 retrieval condition, which did not alter later cue reinstatement, suggesting that reduced lever pressing during retrieval was not coupled to reduced reinstatement.

The underlying mechanism for how memories might be disrupted is unclear. Novelty produces several effects in the brain that can impact attention, motivation, and reward (Schomaker and Meeter, 2015). In our case, the novel VR5 contingency may have re-engaged the mPFC after several days’ experience with the now-familiar, FR1 reinforcement. If nothing new is learned, the mPFC may remain “offline” during FR1 retrieval. The mPFC is involved in rule switching (Cho et al., 2015; Ding et al., 2025; Sohal et al., 2009), and a decrease in PV cell activation in the mPFC during retrieval may have compromised the ability to switch rules under the novel contingency of the VR5 session, impairing updating of the cocaine memory. Retrieval under novel conditions, potentially combined with the presence of cocaine, which destabilizes the PFC network (Borzou et al., 2024), may create instability in the mPFC circuit required for reconsolidation. Supporting this idea, we previously found that the novel VR5 reactivation session activated PV-dependent high frequency oscillatory (gamma) activity in the mPFC that was absent on the last day of training for cocaine self-administration. Moreover, it reduced the coupling of theta-gamma oscillations between the dorsal hippocampus and mPFC (Wingert et al., 2024). Other studies have found that novel stimuli presented after familiar stimuli destabilized neurons in the visual cortex (Nitzan et al., 2024), and disrupted the grid cell network for spatial memory (Christensen et al., 2021). Ketamine may induce a similar disruption of synchrony between brain regions; but it remains to be tested whether this is the case, and what other brain regions contribute to the effect when ketamine is given systemically.

Alternative interpretations to a ketamine-induced impairment of reconsolidation must also be considered. Ketamine may suppress motivation to press for cocaine-associated cues 24 h later, although this explanation *does not preclude* a partial effect of ketamine on memory reconsolidation, since memory would need to be preserved for the motivated behavior to be expressed. A few studies have been conducted in humans and non-human primates that combined ketamine treatment with presentation of a prediction error, similar to the current study. In individuals with alcohol use disorder, a ketamine infusion delivered without memory retrieval reduced alcohol consumption and craving, but not to the same extent as ketamine plus retrieval (Das et al., 2019). In cocaine self-administering monkeys, ketamine administered after a cocaine-induced reinstatement test reduced cocaine self-administration 48 h later (Maltbie et al., 2019). Additionally, in sucrose self-administering rats, subanesthetic ketamine administration prior to a test in which a prediction error was induced by changing timing of an expected reward delivery diminished the motivational value of sucrose cues, and this effect was maintained the following day (Fitzpatrick and Morrow, 2017). Thus, the state induced by pre-retrieval ketamine combined with a prediction error may have diminished motivation to seek cocaine and/or the salience of the cocaine-associated cue.

### 4.2. Ketamine reduces PNNs and c-fos-activated PV cells

We measured the intensity and number of PNNs and PV in the mPFC following cue reinstatement because previous studies have shown that repeated ketamine treatment reduced PNNs in both the somatosensory cortex (Venturino et al., 2021a) and mPFC (Matuszko et al., 2017), although no changes were found in the visual cortex (Cannarozzo et al., 2023). Ketamine has also been shown to reduce PNNs (Venturino et al., 2021b), PV levels, and PV cell firing (Picard et al., 2019; Zhou et al., 2015). We therefore assessed the status of PNNs and PV cells after cue reinstatement in animals treated with ketamine prior to or after cocaine memory retrieval. While there were no effects of the single-dose ketamine on PNN intensity or number of PNNs, ketamine decreased the intensity of PNNs that surrounded c-Fos-activated cells. However, this reduced PNN intensity after pre-retrieval ketamine treatment did not appear to be related to the subsequent reduction in cue reinstatement, because PNN intensity was also reduced when ketamine was delivered post-retrieval, which did not alter cue reinstatement. The mechanism by which ketamine reduces PNNs is not well understood, but one study using repeated, anesthetic doses of ketamine demonstrated microglia-induced degradation of PNNs (Venturino et al., 2021b), and a second study using repeated, subanesthetic doses of ketamine reported changes in the morphology of PNNs (Kaushik et al., 2021). While the ketamine-induced decrease in PNN intensity does not appear to be directly related to reinstatement behavior, ketamine is expected to induce metaplasticity (Nardou et al., 2023), which may share similarities with the impact of reductions in PNN intensity (Sakata et al., 2025) on drug cue reinstatement.

Given the ketamine-induced decrease in PNN intensity after cue reinstatement, it was suprising that ketamine did not alter PV cell number or intensity at this time, since PNN and PV expression typically coincide (Slaker et al., 2018b). It is possible that we may have missed rapid, temporal changes in PV levels, which were measured 24 h later, after ketamine and metabolites are metabolized (Saland and Kabbaj, 2018; Zanos et al., 2016). Indeed, given the extremely rapid (30 min) reduction in PV levels reported after acute, low-dose ketamine (Zhou et al., 2015), we measured PV cell activation using c-Fos as a marker for PV cell activity. The pronounced reduction in number of c-Fos-activated PV cells after novel VR5 retrieval suggests that decreased PV cell function in the mPFC and/or elsewhere in the brain during retrieval may suppress cocaine cue reinstatement. A caveat in our interpretation is that we did not assess c-Fos/PV co-labeled cells immediately after the FR1 retrieval. Moreover, we recognize that the results demonstrating reduced c-Fos co-labeling with PV is one readout of PV cell activation, but this decreased c-Fos does not necessarily correlate with reduced functioning of PV cells. Future studies would need to assess function using slice or *in vivo* electrophysiology. In addition, manipulation of PV neurons using chemo or optogenetics during the memory retrieval time frame would help define the contribution of these neurons in the mPFC to expression of the cocaine-associated memory after cue reinstatement. In the VR5 condition, a decrease in PV cell activation may have led to subsequent reduction in PNNs 24 h later, given the oft-reported bidirectional relationship between PV and PNN expression (Devienne et al., 2021; Favuzzi et al., 2017; Hadler et al., 2024; Yamada et al., 2015).

The major molecular mechanism for reduced PV activation may be due to the ability of ketamine to block NMDA receptors. Most mPFC PV neurons contain NMDA receptors (Lewis et al., 2022), and ketamine rapidly reduces PV cell levels (Zhou et al., 2015) and visual cortical PV cell activity via NMDA receptor blockade (Picard et al., 2019). Ketamine increases mPFC excitability (Gerhard et al., 2020; Moghaddam et al., 1997; Zhang et al., 2021). As a consequence, reduced PV cell activation is expected to lead to a larger number of neurons incorporated into an ensemble, reducing sparse encoding and potentially leading to memory interference (Agetsuma et al., 2018; Lesuis et al., 2025; Morrison et al., 2016). Importantly, feedback excitation of PV neurons is mediated by NMDA receptor signaling that is necessary for maintaining expression of a predominant ensemble that prevails over competing ensembles (Cornford et al., 2019). However, impaired NMDA receptor signaling leads to destabilization of an existing ensemble (Cornford et al., 2019), and ketamine can impair the optimal signal:noise ratio for maintaining memory fidelity (Ma et al., 2015). Thus, the recruitment of additional neurons or destabilization of activated neurons when new information is presented after familiar information (Nitzan et al., 2024) may interfere with the original ensemble that supports the familiar memory. Ketamine given during the processing of new information during the VR5 session may similarly impact memory as that of PNN removal, which reduces PV cell function (Gonzalez et al., 2022; Hanssen et al., 2023; Tewari et al., 2018) and can lead to the loss of specific, sparse ensembles underlying the engram (Ramsaran et al., 2023).

### 4.3. Impact of ketamine on c-Fos and Npas4 levels in cocaine self-administering rats

Since activity of the PFC is necessary for cocaine- and cue-induced reinstatement (Stefanik et al., 2013), we measured a proxy for neuronal activity via expression of the immediate early genes c-Fos and Npas4. While the expression of c-Fos has been found to increase in the mPFC following cocaine cue reinstatement after several extinction days (Kufahl et al., 2009), we did not observe an increase in c-Fos intensity or cell number. This is potentially due to the lack of extensive extinction (30 min in the present study). Overall, our findings with c-Fos intensity indicate that c-Fos was not a sensitive indicator of behavioral output during cue-induced reinstatement.

In contrast to c-Fos, we found that Npas4 intensity was altered after the novel memory retrieval session and/or ketamine treatment. Npas4 is an immediate-early gene and transcription factor that provides excitatory:inhibitory homeostasis on a synaptic and circuit level (Sun and Lin, 2016), and it is selectively and transiently expressed in neurons by synaptic activity and Ca^2+^ influx (Lin et al., 2008). Npas4 expression elicits development and/or maintenance of GABAergic synapses on excitatory neurons (Lin et al., 2008), and it has been implicated in learning and memory (Brito et al., 2024; Ploski et al., 2011; Ramamoorthi et al., 2011; Sun et al., 2020; Sun and Lin, 2016). Npas4 promotes excitation onto inhibitory neurons, thereby inhibiting excitatory output (Spiegel et al., 2014), and its expression in the mPFC plays a major role in chronic stress-induced hypofrontality and anhedonia in mice (Hughes et al., 2023).

While the acute impact of ketamine on adult Npas4 levels has not been described, neonatal exposure to ketamine downregulates Npas4 levels specifically in PV cells in the PFC (Shepard et al., 2019) and is thought to provide homeostatic feedback to the circuit (Spiegel et al., 2014). We demonstrated that ketamine or the novel VR5 retrieval session reduced Npas4 expression in PV cells and PNN-surrounded cells compared with the saline control condition and the familiar FR1 retrieval session. Therefore, the decrease in number of PV and PNN-surrounded Npas4 cells after ketamine in our study suggests that ketamine may dampen this homeostatic feedback and in turn promote mPFC circuit plasticity.

Only a few studies have examined mPFC Npas4 levels after cocaine. *Npas4* mRNA levels are rapidly increased after cocaine in the mPFC (Ye et al., 2016), but we did not assess basal Npas4 levels for comparison, so it is possible that Npas4 was rapidly increased after reinstatement, and that ketamine and the novel retrieval session blunted this response. Although cocaine-associated memory retrieval did not alter Npas4 mRNA in the mPFC of mice after cocaine conditioned place preference (Muniz et al., 2017), decreases in Npas4 mRNA expression in the mPFC were found after daily investigator-delivered cocaine followed by 10 days’ abstinence in adolescent female mice (Berardino et al., 2019). To date, however, there are no studies to our knowledge that have investigated expression of Npas4 in the mPFC of cocaine self-administering rats. Future studies would need to directly test whether manipulating Npas4, particularly within PV cells, would alter reinstatement behavior.

### 4.4. Single-use ketamine reduces methamphetamine-induced, but not fentanyl- or sucrose-induced cue reinstatement

Given that cocaine cue-induced reinstatement was reduced only when a novel retrieval session was used, we employed this protocol when testing the effect of single-use ketamine on reinstatement after self-administration of methamphetamine, fentanyl, or sucrose. Although ketamine given prior to a novel memory retrieval session reduced cue reinstatement in methamphetamine-trained animals, the effects were significant only when comparing within animals, suggesting a reduced magnitude of the effect compared with cocaine self-administering rats. In contrast, no effect of ketamine was apparent in fentanyl or sucrose-trained rats. The lack of lever-pressing during the memory retrieval session in roughly half of the animals trained on fentanyl may have been related to the ability of ketamine or its metabolites to activate μ-opioid receptors (Bonaventura et al., 2021; Joseph et al., 2021) and dampen activity levels on this day. The low responding or lack of responding may have dampened the memory retrieval process, and it is possible that a lower dose of ketamine that does not suppress lever responding during the memory retrieval would reduce subsequent cue reinstatement.

Generally, however, ketamine’s disruptive effect on reinstatement does not appear to be related to performance during the retrieval session. For cocaine- and fentanyl-trained rats, ketamine decreased active lever presses on the retrieval day, which was not observed for methamphetamine- or sucrose-trained rats. We did not measure the effects of the single drug doses of self-administered drugs tested here on brain dopamine levels. However, we suggest based on numerous prior studies that the magnitude of reduction in active lever responding by cocaine-vs. fentanyl-trained rats may be related to the ability of psychostimulants such as cocaine and methamphetamine to elevate dopamine in the nucleus accumbens (Le Cozannet et al., 2013; Pontieri et al., 1995) and/or ketamine-induced increases in PFC excitability and subsequent increases in spine density in the mPFC, which is dependent on dopamine (Wu et al., 2021). Dopamine is increased during prediction error (Schultz, 1998) and promotes learning (Hyman et al., 2006), it promotes lability of memory after retrieval (Merlo et al., 2015) and is a key contributor to overcoming boundary conditions that limit the ability to disrupt reconsolidation (O'Neill and Winters, 2026; Yan et al., 2013). Moreover, dopamine in the mPFC, which is expected to increase during the cocaine-reinforced memory retrieval session, has been shown to drive insertion of calcium permeable AMPARs to promote plasticity (Sun et al., 2005). Finally, in fear learning, PV intensity increases, and this plasticity in PV neurons is dependent on dopamine D1 receptors (Donato et al., 2013; Karunakaran et al., 2016). Thus, increased dopamine presence at the time of memory retrieval in these regions may have facilitated subsequent learning-induced plasticity induced by ketamine. In contrast, sucrose increases nucleus accumbens dopamine with lower magnitude, shorter duration, and slower kinetics than cocaine (Cameron et al., 2014; Lujan et al., 2023), and fentanyl likewise produces a smaller dopaminergic increase overall (Yoshida et al., 1999), which may be insufficient to drive the robust retrieval-dependent plasticity we speculate is associated with psychostimulants. In addition, psychostimulants and opioids also diverge in their actions on the mPFC (Badiani et al., 2011), engaging different neuronal populations (Chang et al., 1998) and producing opposite effects on dendritic spines, with increases in the mPFC after repeated cocaine or amphetamine (Munoz-Cuevas et al., 2013; Robinson and Kolb, 1997), but decreases after repeated opioid (morphine) exposure (Robinson et al., 2002). Moreover, psychostimulants lead to limited plasticity in response to subsequent experience-dependent plasticity (Kolb et al., 2003), which may be reversed by ketamine treatment. Notably, although both drug classes generate silent synapses in the nucleus accumbens (Graziane et al., 2016), they accomplish this through distinct mechanisms: cocaine forms new immature synapses that reopen a plasticity window, whereas opioids remove AMPARs from existing synapses, highlighting that cocaine, opioids, and natural rewards reshape reward circuitry via fundamentally different plasticity processes.

### 4.5. Limitations

One limitation of this study is that we used only single doses of the self-administered drugs and ketamine. Our earlier pilot studies indicated that doses of ketamine (up to 12 mg/kg, ip) would diminish lever pressing during the memory retrieval session, and the dose we chose here (6 mg/kg) still suppressed lever pressing in some groups (lower doses were not tested). Therefore, lower doses of ketamine may also be effective without reducing active lever pressing during retrieval. A second limitation is that ketamine was delivered systemically and thus its effects would impact multiple brain regions, whereas we focused on changes in the mPFC. A third limitation is that the effects of ketamine on cue reinstatement were examined one day later, so it remains untested how long lasting the impact would be. Some human studies have reported long-lasting effects in users of cocaine (Dakwar et al., 2019a; Thomas and Chambers, 2025) and alcohol (Das et al., 2019). Finally, a fourth limitation of this study is that we used only male rats, and sex differences are reported between male and female rats for cocaine and methamphetamine self-administration (Orsini et al., 2022; Reichel et al., 2012) as well as fentanyl (Towers et al., 2022) and sucrose (Grimm et al., 2005, 2022). Female rats, which self-administer at higher rates than males (Orsini et al., 2022) may be less susceptible to the suppressive effects of ketamine. Intriguingly, estrogen receptor-beta is found nearly exclusively within PV neurons in several brain regions (Blurton-Jones and Tuszynski, 2002). Future studies need to examine additional doses of psychostimulants and opioids in both male and female rats.

### 4.6. Conclusions

Our findings suggest that systemic, single-use ketamine treatment given prior to, but not after, a novel memory retrieval session prevented cocaine-associated memory reconsolidation, as measured by reduced cocaine-seeking behavior during cue-induced reinstatement. When administered prior to a familiar retrieval session, ketamine had no impact on subsequent reinstatement. Ketamine combined with a novel retrieval session produced a rapid and transient decrease in the number of c-Fos-activated PV cells in the mPFC, and this decrease in the mPFC and other brain regions may alter mPFC circuit function to reduce memory and/or motivational aspects that drive relapse to the cocaine-associated cue. Ketamine also decreased cue reinstatement in methamphetamine-trained animals, with no effects in fentanyl- or sucrose-trained animals. Our findings offer insights into the potential for innovative approaches to optimize the timing of ketamine administration in the treatment of psychostimulant use disorders.

## Supplementary Material

1

2

3

4

5

6

7

8

9

## Data Availability

Raw data can be obtained from the corresponding author upon reasonable request.

## Appendix A. Supplementary data

Supplementary data to this article can be found online at https://doi.org/10.1016/j.neuropharm.2026.110912.
