# Supplementary figures and images for: Disruption of psychostimulant-associated memories by single, low dose ketamine in rats
Source: Neuropharmacology. Author manuscript; Available in PMC 2026 Jun 12. (PMC13262701; doi:10.1016/j.neuropharm.2026.110912)

Supplemental Fig. 1

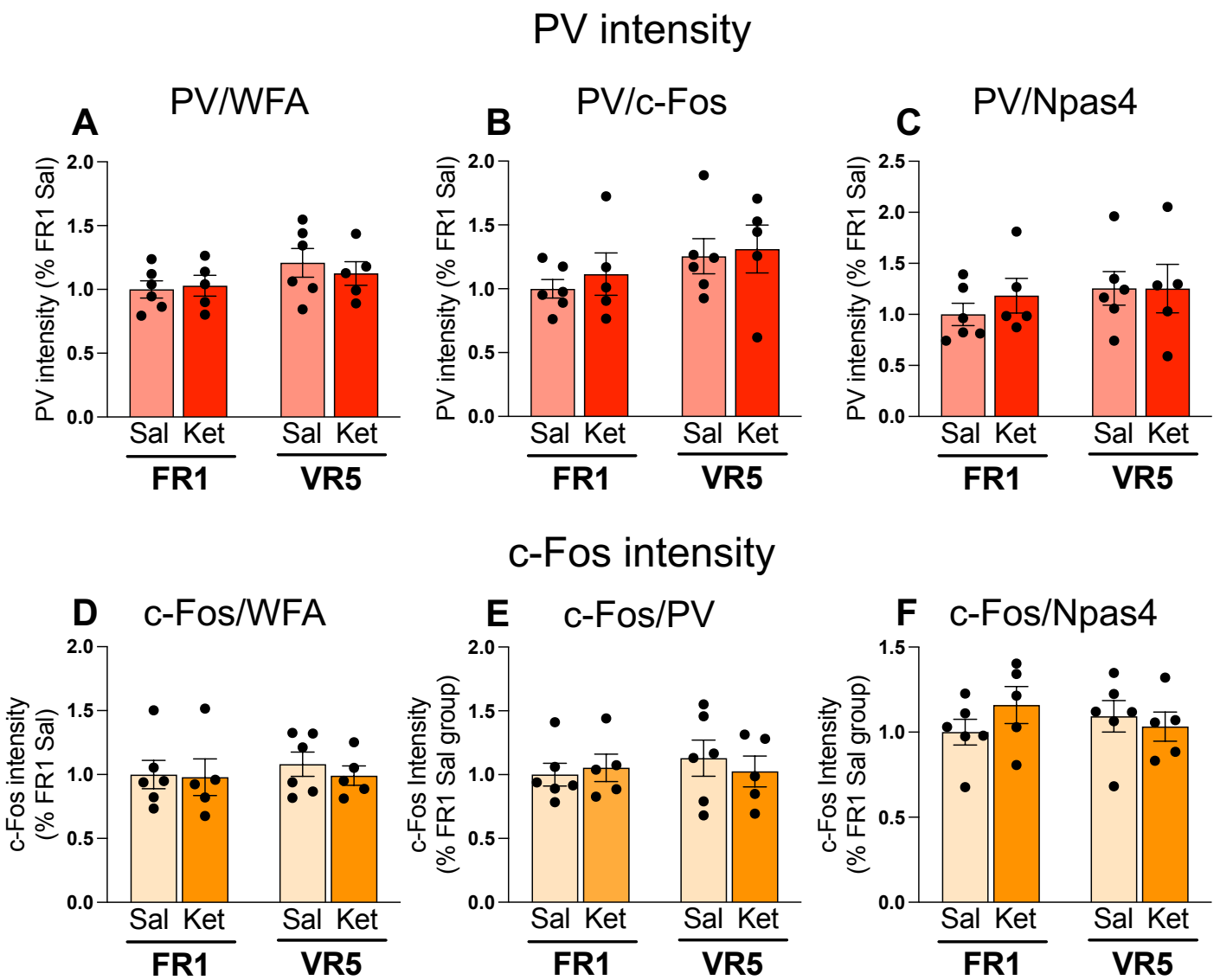

Supplement: 1 [file NIHMS2180145-supplement-1.pdf]
