# Supplementary material for: Disruption of psychostimulant-associated memories by single, low dose ketamine in rats
Source: Neuropharmacology. Author manuscript; Available in PMC 2026 Jun 12. (PMC13262701; doi:10.1016/j.neuropharm.2026.110912)
Supplement: 2 [file NIHMS2180145-supplement-2.pdf]

Supplemental Table 1: Total lever presses and drug infusions during training days

Figure	Measure	Group	N-size	Total ± SEM	Test	F	p-value
Not Shown	**Pre-Retrieval Cocaine** (Active lever)	FR1 Sal	6	514 ± 102	2-way ANOVA	Treatment (Veh vs Ket) F (1, 33) = 0.1066	p=0.7461
		FR1 Ket	8	614 ± 96		Retrieval F (1, 33) = 0.08924	p=0.7670
		VR5 Sal	10	617 ± 158		Treatment x Retrieval F (1, 33) = 1.519	p=0.2265
		VR5 Ket	13	444 ± 48			
Not Shown	**Pre-Retrieval Cocaine** (Infusions)	FR1 Sal	6	354 ± 52	2-way ANOVA	Treatment (Veh vs Ket) F (1, 33) = 0.2869	p=0.5958
		FR1 Ket	8	426 ± 54		Retrieval F (1, 33) = 0.3762	p=0.5438
		VR5 Sal	10	373 ± 44		Treatment x Retrieval F (1, 33) = 1.076	p=0.3072
		VR5 Ket	13	350 ± 32			
Not Shown	**Pre-Retrieval Cocaine** (Inactive lever)	FR1 Sal	6	40 ± 16	2-way ANOVA	Treatment (Veh vs Ket) F (1, 33) = 1.774	p=0.1920
		FR1 Ket	8	135 ± 60		Retrieval F (1, 33) = 0.5399	p=0.4677
		VR5 Sal	10	66 ± 23		Treatment x Retrieval F (1, 33) = 2.394	p=0.1313
		VR5 Ket	13	59 ± 18			
Not Shown	**Post-Retrieval Cocaine** (Active lever)	FR1 Sal	6	743 ± 84	1-way ANOVA	Treatment F (3, 24) = 1.256	p=0.3117
		FR1 Ket	7	490 ± 66			
		VR5 Sal	8	597 ± 49			
		VR5 Ket	7	943 ± 332			
Not Shown	**Post-Retrieval Cocaine** (Infusions)	FR1 Sal	6	550 ± 45	1-way ANOVA	Treatment F (3, 24) = 1.663	p=0.2015
		FR1 Ket	7	401 ± 40			
		VR5 Sal	8	480 ± 21			
		VR5 Ket	7	469 ± 68			
Not Shown	**Post-Retrieval Cocaine** (Inactive lever)	FR1 Sal	6	86 ± 27	1-way ANOVA	Treatment F (3, 24) = 0.7865	p=0.5132
		FR1 Ket	7	30 ± 7			
		VR5 Sal	8	152 ± 123			
		VR5 Ket	7	249 ± 155			
Not Shown	**Pre-Retrieval Methamphetamine** (Active lever)	VR5 Sal	11	248 ± 51	Welch's t	t=0.1222	p=0.9047
		VR5 Ket	10	241 ± 18			
Not Shown	**Pre-Retrieval Methamphetamine** (Infusions)	VR5 Sal	11	169 ± 29	Welch's t	t=0.3497	p=0.7313
		VR5 Ket	10	180 ± 16			
Not Shown	**Pre-Retrieval Methamphetamine** (Inactive lever)	VR5 Sal	11	63 ± 13	Welch's t	t=0.9961	p=0.3452
		VR5 Ket	10	560 ± 499			
Not Shown	**Pre-Retrieval Fentanyl** (Active lever)	VR5 Sal	11	331 ± 39	Welch's t	t=0.2903	p=0.7746
		VR5 Ket	11	315 ± 39			
Not Shown	**Pre-Retrieval Fenatanyl** (Infusions)	VR5 Sal	11	237 ± 26	Welch's t	t=0.3696	p=0.7156
		VR5 Ket	11	224 ± 23			
Not Shown	**Pre-Retrieval Fentanyl** (Inactive lever)	VR5 Sal	11	65 ± 17	Welch's t	t=0.7260	p=0.4763
		VR5 Ket	11	48 ± 16			
Not Shown	**Pre-Retrieval Sucrose** (Active lever)	VR5 Sal	8	1879 ± 228	Welch's t	t=0.2815	p=0.7825
		VR5 Ket	8	1782 ± 260			
Not Shown	**Pre-Retrieval Sucrose** (Infusions)	VR5 Sal	8	1068 ± 78	Welch's t	t=0.0921	p=0.9280
		VR5 Ket	8	1079 ± 98			
Not Shown	**Pre-Retrieval Sucrose** (Inactive lever)	VR5 Sal	8	276 ± 60	Welch's t	t=0.7729	p=0.4576
		VR5 Ket	8	225 ± 28			
