# Supplementary material for: Disruption of psychostimulant-associated memories by single, low dose ketamine in rats
Source: Neuropharmacology. Author manuscript; Available in PMC 2026 Jun 12. (PMC13262701; doi:10.1016/j.neuropharm.2026.110912)
Supplement: 3 [file NIHMS2180145-supplement-3.pdf]

Supplemental Table 2: Figure 2 Cocaine Pre-FR1 or VR5 Retrieval Statistics

Figure	Measure	Group	N-size	Test	F	p-value	Šídák's	p-value
2A	Cocaine Training (Active lever)	FR1 Sal	6	3-way RM ANOVA	Treatment (Veh vs Ket) F (1, 33) = 0.0580	p=0.811		
		FR1 Ket	8		Retrieval F (1,33) = 0.0003	p=0.986		
		VR5 Sal	10		Day F (1.48, 48.91) = 2.531	p=0.104		
		VR5 Ket	13		Treatment x Retrieval F (1, 33) = 1.534	p=0.224		
					Treatment x Day F (1.48, 48.91) = 0.684	p=0.467		
					Retrieval x Day F (1.48, 48.91) = 1.073	p=0.333		
					Treatment x Retrieval x Day F (1.48, 48.91) = 1.08[illegible]	p=0.330		
2B	Cocaine Training (Infusions)	FR1 Sal	6	3-way RM ANOVA	Treatment (Veh vs Ket) F (1, 33) = 0.589	p=0.448		
		FR1 Ket	8		Retrieval F (1,33) = 0.000744	p=0.978		
		VR5 Sal	10		Day F (4.23, 139.54) = 30.49	**p<0.0001**		
		VR5 Ket	13		Treatment x Retrieval F(1, 33) = 1.259	p=0.270		
					Treatment x Day F(4.23, 139.54) = 0.313	p=0.878		
					Retrieval x Day F (4.23, 139.54) = 1.906	p=0.109		
					Treatment x Retrieval x Day F (4.23, 139.54) = 0.6[illegible]	p=0.615		
2C	Cocaine Training (Inactive lever)	FR1 Sal	6	3-way RM ANOVA	Treatment (Veh vs Ket) F (1, 33) = 1.806	p=0.188		
		FR1 Ket	8		Retrieval F (1,33) = 0.452	p=0.506		
		VR5 Sal	10		Day F (1.99, 65.56) = 1.561	p=0.218		
		VR5 Ket	13		Treatment x Retrieval F(1, 33) = 2.416	p=0.130		
					Treatment x Day F (1.99, 65.56) = 0.785	p=0.459		
					Retrieval x Day F (1.99, 65.56) = 0.690	p=0.504		
					Treatment x Retrieval x Day F (1.99, 65.56) = 1.10[illegible]	p=0.338		
2D	Memory Retrieval (Active lever)	FR1 Sal	6	2-way ANOVA	Treatment (Veh vs Ket) F (1, 33) = 5.510	**p=0.0251**	Sal vs Ket (FR1)	p=0.3330
		FR1 Ket	8		Retrieval F (1, 33) = 2.018	p=0.1648	Sal vs Ket (VR5)	p=0.0896
		VR5 Sal	10		Treatment x Retrieval F (1, 33) = 0.0411	p=0.8407		
		VR5 Ket	13					
2E	Memory Retrieval (Infusions)	FR1 Sal	6	2-way ANOVA	Treatment (Veh vs Ket) F (1, 33) = 5.677	**p=0.0231**	Sal vs Ket (FR1)	p=0.1115
		FR1 Ket	8		Retrieval F (1, 33) = 31.10	**p<0.0001**	Sal vs Ket (VR5)	p=0.3380
		VR5 Sal	10		Treatment x Retrieval F (1, 33) = 0.5245	p=0.4740	FR1 vs VR5 (Sal)	**p=0.0004**
		VR5 Ket	13				FR1 vs VR5 (Ket)	**p=0.0016**
Not Shown	Memory Retrieval (Inactive lever)	FR1 Sal	6	2-way ANOVA	Treatment (Veh vs Ket) F (1, 33) = 3.017	p=0.0917		
		FR1 Ket	8		Retrieval F (1, 33) = 0.1383	p=0.7124		
		VR5 Sal	10		Treatment x Retrieval F (1, 33) = 0.0782	p=0.7815		
		VR5 Ket	13					
2F	Extinction (FR1 only) (Active lever)	FR1 Sal	6	2-way RM ANOVA	Treatment F (1, 12) = 0.8808	p=0.3665		
		FR1 Ket	8		Time F (3.687, 44.24) = 17.34	**p<0.0001**		
					Treatment x Time F (5, 60) = 2.039	p=0.0859		
Not Shown	Extinction (FR1 only) (Inactive lever)	FR1 Sal	6	2-way RM ANOVA	Treatment F (1, 12) = 0.09527	p=0.7629		
		FR1 Ket	8		Time F (2.105, 25.26) = 4.258	**p=0.0239**		
					Treatment x Time F (5, 60) = 1.113	p=0.3632		

Supplemental Table 2: Figure 2 Cocaine Pre-FR1 or VR5 Retrieval Statistics - con't								
2G	Extinction (VR5 only) (Active lever)	VR5 Sal	6	2-way RM ANOVA	Treatment F (1, 13) = 4.236	p=0.0602		
		VR5 Ket	9		Time F (3.532, 45.91) = 14.86	**p<0.0001**		
					Treatment x Time F (5, 65) = 0.7608	p=0.5814		
Not Shown	Extinction (VR5 only) (Inactive lever)	VR5 Sal	6	2-way RM ANOVA	Treatment F (1, 13) = 2.279	p=0.1550		
		VR5 Ket	9		Time F (3.432, 44.62) = 2.427	p=0.0703		
					Treatment x Time F (5, 65) = 0.7818	p=0.5665		
2H	Cue Reinstatement (Active lever)	FR1 Sal	6	2-way ANOVA	Treatment (Veh vs Ket) F (1, 25) = 3.858	p=0.0607	Sal vs Ket (FR1)	p=0.9541
		FR1 Ket	8		Retrieval F (1, 25) = 0.7129	p=0.4065	Sal vs Ket (VR5)	**p=0.0096**
		VR5 Sal	6		Treatment x Retrieval F (1, 25) = 5.557	**p=0.0265**	FR1 vs VR5 (Sal)	p=0.5541
		VR5 Ket	9				FR1 vs VR5 (Ket)	**p=0.0395**
2I	Cue Reinstatement Time Course FR1 only (Active lever)	FR1 Sal	6	2-way RM ANOVA	Treatment (Veh vs Ket) F (1, 12) = 0.06801	p=0.7987		
		FR1 Ket	8		Time F (3.090, 37.08) = 5.646	**p=0.0025**		
					Treatment x Time F (5, 60) = 0.6876	p=0.6347		
2J	Cue Reinstatement Time Course VR5 only (Active lever)	FR1 Sal	6	2-way RM ANOVA	Treatment (Veh vs Ket) F (1, 13) = 10.64	**p=0.0062**		
		FR1 Ket	9		Time F (2.702, 35.12) = 7.067	**p=0.0011**		
					Treatment x Time F (5, 65) = 1.998	p=0.0906		
2K	Cue Reinstatement % last 5 d avg (Active lever)	FR1 Sal	6	2-way ANOVA	Treatment (Veh vs Ket) F (1, 25) = 4.505	**p=0.0449**	Sal vs Ket (FR1)	p=0.9978
		FR1 Ket	8		Retrieval F (1, 25) = 0.6155	p=0.4401	Sal vs Ket (VR5)	**p=0.0094**
		VR5 Sal	6		Treatment x Retrieval F (1, 25) = 4.878	**p=0.0366**	FR1 vs VR5 (Sal)	p=0.5906
		VR5 Ket	9				FR1 vs VR5 (Ket)	p=0.0563
2L	Cue Reinstatement (Cue Rewards)	FR1 Sal	6	2-way ANOVA	Treatment (Veh vs Ket) F (1, 25) = 2.506	p=0.1260		
		FR1 Ket	8		Retrieval F (1, 25) = 0.9300	p=0.3441		
		VR5 Sal	6		Treatment x Retrieval F (1, 25) = 3.606	p=0.0692		
		VR5 Ket	9					
2M	Cue Reinstatement Time course FR1 only (Cue Rewards)	VR5 Sal	6	2-way RM ANOVA	Treatment F (1, 12) = 0.03546	p=0.8538		
		VR5 Ket	8		Time F (3.166, 37.99) = 6.515	**p=0.0010**		
					Treatment x Time F (5, 60) = 1.624	p=0.1674		
2N	Cue Reinstatement Time course VR5 only (Cue Rewards)	VR5 Sal	6	2-way RM ANOVA	Treatment F (1, 13) = 9.512	**p=0.0087**		
		VR5 Ket	9		Time F (2.976, 38.69) = 7.891	**p=0.0003**		
					Treatment x Time F (5, 65) = 1.837	p=0.1179		
2O	Cue Reinstatement % last 5 d avg (Cue rewards)	FR1 Sal	6	2-way ANOVA	Treatment F (1, 25) = 7.280	**p=0.0123**	Sal vs Ket (FR1)	p=0.6725
		FR1 Ket	8		Retrieval F (1, 25) = 0.9175	p=0.3473	Sal vs Ket (VR5)	**p=0.0110**
		VR5 Sal	6		Treatment x Retrieval F (1, 25) = 2.384	p=0.1351		
		VR5 Ket	9					
Not Shown	Cue Reinstatement (Inactive lever)	FR1 Sal	6	2-way ANOVA	Treatment F (1, 25) = 0.8620	p=0.3621		
		FR1 Ket	8		Retrieval F (1, 25) = 4.023	p=0.0558		
		VR5 Sal	6		Treatment x Retrieval F (1, 25) = 1.503	p=0.2316		
		VR5 Ket	9					
