# Supplementary material for: Disruption of psychostimulant-associated memories by single, low dose ketamine in rats
Source: Neuropharmacology. Author manuscript; Available in PMC 2026 Jun 12. (PMC13262701; doi:10.1016/j.neuropharm.2026.110912)
Supplement: 4 [file NIHMS2180145-supplement-4.pdf]

Supplemental Table 3: Figure 3 Cocaine Post-VR5 Retrieval Statistics

Figure	Measure	Group	N-size	Test	F	p-value	Dunnett's	p-value
3A	Cocaine Training (Active lever)	VR5 Sal	6	2-way RM ANOVA	Treatment (Veh vs Ket) F (3, 24) = 1.453	p=0.2524		
		VR5 Ket 6	7		Day F (1.938, 46.52) = 0.9971	p=0.3745		
		VR5 Ket 20	8		Treatment x Day F (36, 288) = 1.492	**p=0.0404**		
		VR5 Ket 50	7					
3B	Cocaine. Training (Infusions)	VR5 Sal	6	2-way RM ANOVA	Treatment (Veh vs Ket) F (3, 24) = 1.679	p=0.1981	Day 6:Sal vs 6 Ket	p=0.0460
		VR5 Ket 6	7		Day F (4.416, 106.0) = 21.24	**p< 0.0001**	Day 13:Sal vs 6 Ket	**p=0.0086**
		VR5 Ket 20	8		Treatment x Day F (36, 288) = 1.702	**p=0.0097**		
		VR5 Ket 50	7					
3C	Cocaine Training (Inactive lever)	VR5 Sal	6	2-way RM ANOVA	Treatment (Veh vs Ket) F (3, 24) = 0.7895	p=0.5116		
		VR5 Ket 6	7		Day F (1.463, 35.10) = 1.408	p=0.2542		
		VR5 Ket 20	8		Treatment x Day F (36, 288) = 0.7779	p=0.8172		
		VR5 Ket 50	7					
3D	Memory Retrieval (Active lever)	VR5 Sal	6	1-way ANOVA	Treatment F (3, 24) = 2.795	p=0.0620		
		VR5 Ket 6	7					
		VR5 Ket 20	8					
		VR5 Ket 50	7					
3E	Memory Retrieval (Infusions)	VR5 Sal	6	1-way ANOVA	Treatment F (3, 24) = 2.830	p=0.0598		
		VR5 Ket 6	7					
		VR5 Ket 20	8					
		VR5 Ket 50	7					
Not Shown	Memory Retrieval (Inactive lever)	VR5 Sal	6	1-way ANOVA	Treatment F (3, 24) = 4.311	**p=0.0144**		
		VR5 Ket 6	7					
		VR5 Ket 20	8					
		VR5 Ket 50	7					
3F	Extinction (Active lever)	VR5 Sal	6	2-way RM ANOVA	Treatment F (3, 24) = 0.4362	p=0.7291		
		VR5 Ket 6	7		Time F (3.792, 91.01) = 17.18	**p<0.0001**		
		VR5 Ket 20	8		Treatment x Time F (15, 120) = 1.647	p=0.0713		
		VR5 Ket 50	7					
Not Shown	Extinction (Inactive lever)	VR5 Sal	6	2-way RM ANOVA	Treatment F (3, 24) = 1.115	p=0.3625		
		VR5 Ket 6	7		Time F (3.162, 75.89) = 2.318	p=0.0790		
		VR5 Ket 20	8		Treatment x Time F (15, 120) = 0.9752	p=0.4854		
		VR5 Ket 50	7					
3G	Cue Reinstatement (Active lever)	VR5 Sal	6	1-way ANOVA	Treatment F (3, 24) = 1.119	p=0.3609		
		VR5 Ket 6	7					
		VR5 Ket 20	8					
		VR5 Ket 50	7					
3H	Cue Reinstatement Time course (Active lever)	VR5 Sal	6	2-way RM ANOVA	Treatment F (3, 24) = 1.119	p=0.3609		
		VR5 Ket 6	7		Time F (3.555, 85.32) = 7.846	**p<0.0001**		
		VR5 Ket 20	8		Treatment x Time F (15, 120) = 0.5501	p=0.9065		
		VR5 Ket 50	7					
3I	Cue Reinstatement (Cue Rewards)	VR5 Sal	6	1-way ANOVA	Treatment F (3, 24) = 1.860	p=0.1634		
		VR5 Ket 6	7					
		VR5 Ket 20	8					
		VR5 Ket 50	7					
3J	Cue Reinstatement Time course (Cue Rewards)	VR5 Sal	6	2-way RM ANOVA	Treatment F (3, 24) = 1.860	p=0.1634		
		VR5 Ket 6	7		Time F (3.466, 83.19) = 9.238	**p<0.0001**		
		VR5 Ket 20	8		Treatment x Time F (15, 120) = 0.5646	p=0.8966		
		VR5 Ket 50	7					
Not Shown	Cue Reinstatement (Inactive lever)	VR5 Sal	6	1-way ANOVA	Treatment F (3, 24) = 1.161	p=0.3450		
		VR5 Ket 6	7					
		VR5 Ket 20	8					
		VR5 Ket 50	7					
