# Supplementary material for: Disruption of psychostimulant-associated memories by single, low dose ketamine in rats
Source: Neuropharmacology. Author manuscript; Available in PMC 2026 Jun 12. (PMC13262701; doi:10.1016/j.neuropharm.2026.110912)
Supplement: 5 [file NIHMS2180145-supplement-5.pdf]

Supplemental Table 4: Figure 4 and Supplemental Figure 1 Statistics

Figure	Measure	Group	N-size	Test	F	p-value	Šídák's	p-value
4F	**Pre-Retrieval Ket:** WFA Intensity around PV cells	FR1 Sal	6	2-way ANOVA	Treatment (Veh vs Ket) F (1, 18) = 3.386	p=0.0823		
		FR1 Ket	5		Retrieval F (1, 18) = 1.601	p=0.2219		
		VR5 Sal	6		Treatment x Retrieval F (1, 18) = 0.2167	p=0.6471		
		VR5 Ket	5					
4G	**Pre-Retrieval Ket:** WFA Intensity around c-Fos cells	FR1 Sal	6	2-way ANOVA	Treatment (Veh vs Ket) F (1, 18) = 6.018	**p=0.0246**	Sal vs Ket (FR1)	p=0.6959
		FR1 Ket	5		Retrieval F (1, 18) = 2.588	p=0.1251	Sal vs Ket (VR5)	**p=0.0294**
		VR5 Sal	6		Treatment x Retrieval F (36, 288) = 1.702	p=0.1914		
		VR5 Ket	5					
4H	**Pre-Retrieval Ket:** WFA Intensity around NPAS4 cells	FR1 Sal	6	2-way ANOVA	Treatment (Veh vs Ket) F (1, 18) = 1.964	p=0.1781		
		FR1 Ket	5		Retrieval F (1, 18) = 3.426	p=0.0807		
		VR5 Sal	6		Treatment x Retrieval F (1, 18) = 0.0002880	p=0.9866		
		VR5 Ket	5					
4I	**Pre-Retrieval Ket:** NPAS4 Intensity in PV cells	FR1 Sal	6	2-way ANOVA	Treatment (Veh vs Ket) F (1, 18) = 6.185	**p=0.0229**	Sal vs Ket (FR1)	**p=0.0318**
		FR1 Ket	5		Retrieval F (1, 18) = 1.689	p=0.2102	Sal vs Ket (VR5)	p=0.6421
		VR5 Sal	6		Treatment x Retrieval F (1, 18) = 1.619	p=0.2194		
		VR5 Ket	5					
4J	**Pre-Retrieval Ket:** NPAS4 Intensity in c-Fos cells	FR1 Sal	6	2-way ANOVA	Treatment (Veh vs Ket) F (1, 18) = 3.033	p=0.0986		
		FR1 Ket	5		Retrieval F (1, 18) = 1.484	p=0.2389		
		VR5 Sal	6		Treatment x Retrieval F (1, 18) = 1.461	p=0.2425		
		VR5 Ket	5					
4K	**Pre-Retrieval Ket:** NPAS4 Intensity in WFA surrounded cells	FR1 Sal	6	2-way ANOVA	Treatment (Veh vs Ket) F (1, 18) = 1.932	p=0.1815		
		FR1 Ket	5		Retrieval F (1, 18) = 4.901	**p=0.0400**		
		VR5 Sal	6		Treatment x Retrieval F (1, 18) = 1.990	p=0.1754	FR1 vs VR5 (Sal)	**p=0.0298**
		VR5 Ket	5				FR1 vs VR5 (Ket)	p=0.8346
Supp Fig 1A	**Pre-Retrieval Ket:** PV Intensity in WFA surrounded cells	FR1 Sal	6	2-way ANOVA	Treatment F (1, 18) = 0.08769	p=0.7705		
		FR1 Ket	5		Retrieval F (1, 18) = 2.770	p=0.1134		
		VR5 Sal	6		Treatment x Retrieval F (1, 18) = 0.3785	p=0.5461		
		VR5 Ket	5					
Supp Fig 1B	**Pre-Retrieval Ket:** PV Intensity in c-Fos cells	FR1 Sal	6	2-way ANOVA	Treatment F (1, 18) = 0.3657	p=0.5529		
		FR1 Ket	5		Retrieval F (1, 18) = 2.523	p=0.1296		
		VR5 Sal	6		Treatment x Retrieval F (1, 18) = 0.04199	p=0.8399		
		VR5 Ket	5					
Supp Fig 1C	**Pre-Retrieval Ket:** PV Intensity in NPAS4 cells	FR1 Sal	6	2-way ANOVA	Treatment F (1, 18) = 0.2828	p=0.6014		
		FR1 Ket	5		Retrieval F (1, 18) = 0.8757	p=0.3618		
		VR5 Sal	6		Treatment x Retrieval F (1, 18) = 0.2945	p=0.5940		
		VR5 Ket	5					
Supp Fig 1D	**Pre-Retrieval Ket:** c-Fos Intensity in WFA surrounded cells	FR1 Sal	6	2-way ANOVA	Treatment F (1, 18) = 0.2575	p=0.6180		
		FR1 Ket	5		Retrieval F (1, 18) = 0.1822	p=0.6745		
		VR5 Sal	6		Treatment x Retrieval F (1, 18) = 0.09850	p=0.7572		
		VR5 Ket	5					
Supp Fig 1E	**Pre-Retrieval Ket:** c-Fos Intensity in PV cells	FR1 Sal	6	2-way ANOVA	Treatment F (1, 18) = 0.04601	p=0.8326		
		FR1 Ket	5		React F (1, 18) = 0.1840	p=0.6731		
		VR5 Sal	6		Treatment x React F (1, 18) = 0.4477	p=0.5119		
		VR5 Ket	5					
Supp Fig 1F	**Pre-Retrieval Ket:** c-Fos Intensity in NPAS4 cells	FR1 Sal	6	2-way ANOVA	Treatment F (1, 18) = 0.2961	p=0.5930		
		FR1 Ket	5		React F (1, 18) = 0.03260	p=0.8587		
		VR5 Sal	6		Treatment x React F (1, 18) = 1.457	p=0.2431		
		VR5 Ket	5					
Figure 4N	**Post-Retrieval Ket: Cue Reinstatement** WFA Intensity around PV cells	VR5 Sal	6	Welch's t	t=5.850	**p< 0.0001**		
		VR5 Ket	7					
Not shown	**Pre-Retrieval Ket: Retrieval** WFA Intensity around PV cells	VR5 Sal	6	Welch's t	t=0.7692	p=0.4728		
		VR5 Ket	7					
Figure 4Q	**Pre-Retrieval Ket: Retrieval** c-Fos/PV cell number	VR5 Sal	4	Welch's t	t=5.641	**p=0.0014**		
		VR5 Ket	4					
