# Supplementary material for: Disruption of psychostimulant-associated memories by single, low dose ketamine in rats
Source: Neuropharmacology. Author manuscript; Available in PMC 2026 Jun 12. (PMC13262701; doi:10.1016/j.neuropharm.2026.110912)
Supplement: 6 [file NIHMS2180145-supplement-6.pdf]

Supplemental Table 5: Cell numbers for Pre-FR1/VR5 Retrieval Statistics

Figure	Measure	Group	N-size	Cell # ± SEM	Test	F	p-value
Not shown	**Pre-Retrieval Ket:** WFA/PV cell number	FR1 Sal	6	3.5 ± 0.4	2-way ANOVA	Treatment (Veh vs Ket) F (1, 18) = 0.7242	p=0.4059
		FR1 Ket	5	3.9 ± 0.5		Retrieval F (1, 18) = 0.6014	p=0.4481
		VR5 Sal	6	3.9 ± 0.6		Treatment x Retrieval F (1, 18) = 0.006150	p=0.9384
		VR5 Ket	5	4.4 ± 0.7			
Not shown	**Pre-Retrieval Ket:** WFA/c-Fos cell number	FR1 Sal	6	4.1 ± 0.5	2-way ANOVA	Treatment (Veh vs Ket) F (1, 18) = 0.1548	p=0.6986
		FR1 Ket	5	3.8 ± 2.8		Retrieval F (1, 18) = 1.148	p=0.2981
		VR5 Sal	6	3.1 ± 0.3		Treatment x Retrieval F (1, 18) = 1.412	p=0.2502
		VR5 Ket	5	3.8 ± 0.6			
Not shown	**Pre-Retrieval Ket:** WFA/NPAS4 cell number	FR1 Sal	6	3.2 ± 0.3	2-way ANOVA	Treatment (Veh vs Ket) F (1, 18) = 0.1125	p=0.7412
		FR1 Ket	5	3.2 ± 0.4		Retrieval F (1, 18) = 3.900	p=0.0638
		VR5 Sal	6	2.3 ± 0.5		Treatment x Retrieval F (1, 18) = 0.06804	p=0.7972
		VR5 Ket	5	2.5 ± 0.4			
Not shown	**Pre-Retrieval Ket:** PV/c-Fos cell number	FR1 Sal	6	4.7 ± 0.4	2-way ANOVA	Treatment (Veh vs Ket) F (1, 18) = 0.08244	p=0.7773
		FR1 Ket	5	5.0 ± 1.0		Retrieval F (1, 18) = 0.2210	p=0.6440
		VR5 Sal	6	5.2 ± 1.0		Treatment x Retrieval F (1, 18) = 0.01953	p=0.8904
		VR5 Ket	5	5.8 ± 2.4			
Not shown	**Pre-Retrieval Ket:** PV/NPAS4 cell number	FR1 Sal	6	4.5 ± 0.6	2-way ANOVA	Treatment (Veh vs Ket) F (1, 18) = 8.448e-032	p>0.9999
		FR1 Ket	5	4.4 ± 0.7		Retrieval F (1, 18) = 0.06258	p=0.8053
		VR5 Sal	6	4.7 ± 1.3		Treatment x Retrieval F (1, 18) = 0.01791	p=0.8950
		VR5 Ket	5	4.8 ± 1.8			
Not shown	**Pre-Retrieval Ket:** c-Fos/NPAS4 cell number	FR1 Sal	6	39.8 ± 4.1	2-way ANOVA	Treatment (Veh vs Ket) F (1, 18) = 0.2814	p=0.6023
		FR1 Ket	5	31.3 ± 6.2		Retrieval F (1, 18) = 0.08837	p=0.7697
		VR5 Sal	6	32.8 ± 7.3		Treatment x Retrieval F (1, 18) = 0.5885	p=0.4529
		VR5 Ket	5	34.4 ± 8.2			
