# Supplementary material for: Disruption of psychostimulant-associated memories by single, low dose ketamine in rats
Source: Neuropharmacology. Author manuscript; Available in PMC 2026 Jun 12. (PMC13262701; doi:10.1016/j.neuropharm.2026.110912)
Supplement: 7 [file NIHMS2180145-supplement-7.pdf]

Supplemental Table 6: Figure 5 Methamphetamine Statistics

Figure	Measure	Group	N-size	Test	F	p-value
5A	Methamphetamine Training (Active lever)	VR5 Sal	11	2-way RM ANOVA	Treatment (Veh vs Ket) F (1, 19) = 0.01379	p=0.9078
		VR5 Ket	10		Day F (3.701, 70.32) = 4.465	**p=0.0036**
					Treatment x Day F (11, 209) = 1.181	p=0.3017
5B	Methamphetamine Training (Infusions)	VR5 Sal	11	2-way RM ANOVA	Treatment (Veh vs Ket) F (1, 19) = 0.1156	p=0.7376
		VR5 Ket	10		Day F (4.283, 81.37) = 11.43	**p< 0.0001**
					Treatment x Day F (11, 209) = 1.530	p=0.1225
5C	MethamphetamineTraining (Inactive lever)	VR5 Sal	11	2-way RM ANOVA	Treatment (Veh vs Ket) F (1, 19) = 1.097	p=0.3081
		VR5 Ket	10		Day F (1.008, 19.15) = 0.9739	p=0.3367
					Treatment x Day F (11, 209) = 1.056	p=0.3989
5D	Memory Retrieval (Active lever)	VR5 Sal	11	Welch's t	t=0.4465	p=0.6604
		VR5 Ket	10			
5E	Memory Retrieval (Infusions)	VR5 Sal	11	Welch's t	t=0.2268	p=0.8231
		VR5 Ket	10			
Not Shown	Memory Retrieval (Inactive lever)	VR5 Sal	11	Welch's t	t=2.124	**p=0.0471**
		VR5 Ket	10			
5F	Extinction (Active lever)	VR5 Sal	11	2-way RM ANOVA	Treatment F (1, 19) = 1.246	p=0.2782
		VR5 Ket	10		Time F (2.856, 54.26) = 16.22	**p<0.0001**
					Treatment x Time F (5, 95) = 2.168	p=0.0641
Not Shown	Extinction (Inactive lever)	VR5 Sal	11	2-way RM ANOVA	Treatment F (1, 19) = 1.881	p=0.1862
		VR5 Ket	10		Time F (2.203, 41.85) = 4.552	**p=0.0138**
					Treatment x Time F (5, 95) = 0.6599	p=0.6548
5G	Cue Reinstatement (Active lever)	VR5 Sal	11	Welch's t	t=1.614	p=0.1257
		VR5 Ket	10			
5H	Cue Reinstatement Time course (Active lever)	VR5 Sal	11	2-way RM ANOVA	Treatment F (1, 19) = 2.480	p=0.1318
		VR5 Ket	10		Time F (2.936, 55.78) = 4.956	**p=0.0043**
					Treatment x Time F (5, 95) = 0.6590	p=0.6554
5I	Cue Reinstatement % last 5 d avg (Active lever)	VR5 Sal	11	Welch's t	t=2.408	**p=0.0264**
		VR5 Ket	10			
5J	Cue Reinstatement (Cue Rewards)	VR5 Sal	11	Welch's t	t=1.527	p=0.1434
		VR5 Ket	10			
5K	Cue Reinstatement Time course (Cue Rewards)	VR5 Sal	11	2-way ANOVA	Treatment F (1, 19) = 0.8211	p=0.3762
		VR5 Ket	10		Time F (3.141, 59.67) = 7.527	**p=0.0002**
					Treatment x Time F (5, 95) = 1.252	p=0.2911
5L	Cue Reinstatement % last 5 d avg (Cue Rewards)	VR5 Sal	11	Welch's t	t=2.457	**p=0.0241**
		VR5 Ket	10			
Not Shown	Cue Reinstatement (Inactive lever)	VR5 Sal	11	Welch's t	t=0.1539	p=0.8796
		VR5 Ket	10			
