# Supplementary material for: Disruption of psychostimulant-associated memories by single, low dose ketamine in rats
Source: Neuropharmacology. Author manuscript; Available in PMC 2026 Jun 12. (PMC13262701; doi:10.1016/j.neuropharm.2026.110912)
Supplement: 8 [file NIHMS2180145-supplement-8.pdf]

Supplemental Table 7: Figure 6 Fentanyl Statistics

Figure	Measure	Group	N-size	Test	F	p-value
6A	Fentanyl Training (Active lever)	VR5 Sal	11	2-way RM ANOVA	Treatment (Veh vs Ket) F (1, 20) = 0.2545	p=0.6195
		VR5 Ket	11		Day F (5.275, 99.79) = 5.756	**p< 0.0001**
					Treatment x Day F (12, 227) = 0.8394	p=0.6097
6B	Fentanyl Training (Infusions)	VR5 Sal	11	2-way RM ANOVA	Treatment (Veh vs Ket) F (1, 20) = 0.3444	p=0.5639
		VR5 Ket	11		Day F (4.589, 86.82) = 9.210	**p< 0.0001**
					Treatment x Day F (12, 227) = 0.7467	p=0.7046
6C	Fentanyl Training (Inactive lever)	VR5 Sal	11	2-way RM ANOVA	Treatment (Veh vs Ket) F (1, 20) = 0.3833	p=0.5428
		VR5 Ket	11		Day F (4.205, 77.45) = 0.7323	p=0.5791
					Treatment x Day F (12, 221) = 1.087	p=0.3724
6D	Memory Retrieval (Active lever)	VR5 Sal	11	Welch's t	t=3.020	**p=0.0101**
		VR5 Ket	11			
6E	Memory Retrieval (Infusions)	VR5 Sal	11	Welch's t	t=3.180	**p=0.0085**
		VR5 Ket	11			
Not Shown	Memory Retrieval (Inactive lever)	VR5 Sal	11	Welch's t	t=1.362	p=0.1883
		VR5 Ket	11			
6F	Extinction (Active lever)	VR5 Sal	11	2-way RM ANOVA	Treatment F (1, 16) = 0.4264	p=0.5230
		VR5 Ket	11		Time F (3.065, 49.04) = 12.66	**p<0.0001**
					Treatment x Time F (3.065, 49.04) = 1.466	p=0.2347
Not Shown	Extinction (Inactive lever)	VR5 Sal	11	2-way RM ANOVA	Treatment F (1, 16) = 0.02817	p=0.8688
		VR5 Ket	11		Time F (3.155, 50.48) = 1.551	p=0.2112
					Treatment x Time F (3.155, 50.48) = 1.088	p=0.3645
6G	Cue Reinstatement (Active lever)	VR5 Sal	11	Welch's t	t=0.6689	p=0.5134
		VR5 Ket	11			
6H	Cue Reinstatement Time course (Active lever)	VR5 Sal	11	2-way RM ANOVA	Treatment F (1, 20) = 0.4474	p=0.5112
		VR5 Ket	11		Time F (3.708, 74.15) = 7.717	**p<0.0001**
					Treatment x Time F (3.708, 74.15) = 0.7816	p=0.5320
6I	Cue Reinstatement % last 5 d avg (Active lever)	VR5 Sal	11	Welch's t	t=0.1640	p=0.8715
		VR5 Ket	11			
6J	Cue Reinstatement (Cue Rewards)	VR5 Sal	11	Welch's t	t=1.144	p=0.2711
		VR5 Ket	11			
6K	Cue Reinstatement Time course (Cue Rewards)	VR5 Sal	11	2-way RM ANOVA	Treatment F (1, 20) = 1.309	p=0.2661
		VR5 Ket	11		Time F (3.567, 71.34) = 12.84	**p<0.0001**
					Treatment x Time F (3.567, 71.34) = 0.7657	p=0.5375
6L	Cue Reinstatement % last 5 d avg (Cue Rewards)	VR5 Sal	11	Welch's t	t=0.8069	p=0.4299
		VR5 Ket	11			
Not Shown	Cue Reinstatement (Inactive lever)	VR5 Sal	11	Welch's t	t=0.6506	p=0.5237
		VR5 Ket	11			
