# Supplementary material for: Disruption of psychostimulant-associated memories by single, low dose ketamine in rats
Source: Neuropharmacology. Author manuscript; Available in PMC 2026 Jun 12. (PMC13262701; doi:10.1016/j.neuropharm.2026.110912)
Supplement: 9 [file NIHMS2180145-supplement-9.pdf]

**Supplemental Table 8: Figure 7 Sucrose Statistics**

| Figure | Measure | Group | N-size | Test | F | p-value |
|---|---|---|---|---|---|---|
| 7A | Sucrose Training (Active lever ) | VR5 Sal | 8 | 2-way RM ANOVA | Treatment (Veh vs Ket) F (1, 14) = 0.0793 | p=0.7824 |
| | | VR5 Ket | 8 | | Day F (3.203, 44.84) = 1.587 | p=0.2034 |
| | | | | | Treatment x Day F (12, 168) = 0.8340 | p=0.6153 |
| 7B | Sucrose Training (Sucrose pellets) | VR5 Sal | 8 | 2-way RM ANOVA | Treatment (Veh vs Ket) F (1, 14) = 0.00842 | p=0.9279 |
| | | VR5 Ket | 8 | | Day F (3.402, 47.63) = 2.455 | p=0.0674 |
| | | | | | Treatment x Day F (12, 168) = 0.6122 | p=0.8300 |
| 7C | Sucrose Training (Inactive lever) | VR5 Sal | 8 | 2-way RM ANOVA | Treatment (Veh vs Ket) F (1, 14) = 0.5974 | p=0.4524 |
| | | VR5 Ket | 8 | | Day F (4.566, 63.92) = 26.10 | **p<0.0001** |
| | | | | | Treatment x Day F (12, 168) = 1.088 | p=0.3731 |
| 7D | Sucrose Retrieval (Active lever) | VR5 Sal | 8 | Welch's t | t=0.5458 | p=0.5944 |
| | | VR5 Ket | 8 | | | |
| 7E | Sucrose Retrieval (Sucrose pellets) | VR5 Sal | 8 | Welch's t | t=0.2521 | p=0.8046 |
| | | VR5 Ket | 8 | | | |
| Not Shown | Sucrose Retrieval (Inactive lever) | VR5 Sal | 8 | Welch's t | t=2.159 | p=0.0532 |
| | | VR5 Ket | 8 | | | |
| 7F | Extinction (Active lever) | VR5 Sal | 8 | 2-way RM ANOVA | Treatment F (1, 14) = 0.0872 | p=0.7723 |
| | | VR5 Ket | 8 | | Time F (1.945, 27.22) = 18.70 | **p<0.0001** |
| | | | | | Treatment x Time F (1.945, 27.22) = 1.114 | p=0.3414 |
| Not Shown | Extinction (Inactive lever) | VR5 Sal | 8 | 2-way RM ANOVA | Treatment F (1, 14) = 0.04476 | p=0.8355 |
| | | VR5 Ket | 8 | | Time F (2.464, 34.49) = 18.90 | **p<0.0001** |
| | | | | | Treatment x Time F (2.464, 34.49) = 1.722 | p=0.1878 |
| 7G | Cue Reinstatement (Active lever) | VR5 Sal | 8 | Welch's t | t=1.361 | p=0.1993 |
| | | VR5 Ket | 8 | | | |
| 7H | Cue Reinstatement Time course (Active lever) | VR5 Sal | 8 | 2-way RM ANOVA | Treatment F (1, 14) =1.852 | p=0.1950 |
| | | VR5 Ket | 8 | | Time F (3.092, 43.29) = 5.938 | **p=0.0016** |
| | | | | | Treatment x Time F (3.092, 43.29) = 0.7147 | p=0.5525 |
| 7I | Cue Reinstatement % last 5 d avg (Active lever) | VR5 Sal | 8 | Welch's t | t=0.9845 | p=0.3416 |
| | | VR5 Ket | 8 | | | |
| 7J | Cue Reinstatement (Cue Rewards) | VR5 Sal | 8 | Welch's t | t=1.350 | p=0.2038 |
| | | VR5 Ket | 8 | | | |
| 7K | Cue Reinstatement Time course (Cue Rewards) | VR5 Sal | 8 | 2-way RM ANOVA | Treatment F (1, 14) = 1.822 | p=0.1985 |
| | | VR5 Ket | 8 | | Time F (3.399, 47.59) = 4.835 | **p=0.0037** |
| | | | | | Treatment x Time F (3.399, 47.59) = 0.5603 | p=0.6650 |
| 7L | Cue Reinstatement % last 5 d avg (Cue Rewards) | VR5 Sal | 8 | Welch's t | t=1.552 | p=0.1454 |
| | | VR5 Ket | 8 | | | |
| Not Shown | Cue Reinstatement (Inactive lever) | VR5 Sal | 8 | Welch's t | t=1.385 | p=0.1959 |
| | | VR5 Ket | 8 | | | |
